# Remote Measurement-Based Care Interventions for Mental Health: Systematic Review and Meta-Analysis

**DOI:** 10.2196/63088

**Published:** 2026-01-28

**Authors:** Felix Machleid, Twyla Michnevich, Leu Huang, Louisa Schröder-Frerkes, Caspar Wiegmann, Toni Muffel, Jakob Kaminski

**Affiliations:** 1 Department of Psychiatry and Neurosciences, Charité - Universitätsmedizin Berlin, Charitéplatz 1, Berlin, 10117, Germany, 49 30450617297; 2 Berlin Institute of Health at Charité - Universitätsmedizin Berlin, Berlin, Germany; 3 Department of Infectious Diseases and Respiratory Medicine, Charité Campus Virchow-Klinikum, Charité - Universitätsmedizin Berlin, Berlin, Germany; 4 Clinics for Psychiatry and Psychotherapy, Clinics at the Vivantes Klinikum am Urban, Berlin, Germany; 5 Clinic for Psychiatry and Psychotherapy, Clinics at Theodor-Wenzel-Werk e.V., Berlin, Germany; 6 Recovery Cat GmbH, Berlin, Germany

**Keywords:** ecological momentary assessment, RMBC, remote measurement-based care, MMH, mobile mental health, systematic review, meta-analysis, relapse, quality of life, mental disorder, psychological, psychiatric

## Abstract

**Background:**

Poor management of mental health conditions leads to reduced adherence to treatment, prolonged illness, unnecessary rehospitalization, and a significant financial burden to the health care system. Recognizing this, ecological momentary assessment (EMA) and remote measurement-based care (RMBC) interventions have emerged as promising strategies to address gaps in current care systems. They provide a convenient means to continuously monitor patient-reported outcomes, thereby informing clinical decision-making and potentially improving outcomes such as psychopathology, relapse, and quality of life.

**Objective:**

This systematic review and meta-analysis aims to comprehensively appraise and analyze the existing evidence on the use of EMA and RMBC for people living with mental illness.

**Methods:**

The study was conducted according to PRISMA-P (Preferred Reporting Items for Systematic Review and Explanation Meta-Analysis Protocols) guidelines and preregistered with the PROSPERO systematic review registry. A comprehensive search was conducted in 4 online databases using Medical Subject Headings terms related to mental disorders and digital technologies. Studies were included if they included adults with a formally diagnosed mental disorder and measured symptoms using EMA or RMBC. Studies were independently reviewed by subgroups of authors, and data were extracted focusing on symptom-focused or disease-specific outcomes, relapse, recovery-focused outcomes, global functioning, quality of life, and acceptability of the intervention. We performed a descriptive analysis of demographic variables and a meta-analysis of randomized controlled trials (RCTs). Risk of bias was assessed using the Cochrane risk-of-bias tool for randomized trials version 2 (RoB-2).

**Results:**

The systematic review included 103 studies, of which 15 used RMBC. Of these, 9 were RCTs that were meta-analyzed. RMBC interventions varied in effectiveness, generally showing small but significant effects on symptom-specific outcomes, with notable effects on mania symptoms and empowerment. The mean adherence rate across studies to all tracking items was 74.5% (SD 13.98; n=38). More prompts per day, but not more items per prompt, were associated with lower adherence. Adverse effects were infrequently reported and included technical problems and psychological distress. Concerns about bias were raised, particularly regarding participants’ awareness of the interventions and potential deviations from the intended protocols.

**Conclusions:**

Although RMBC shows growing potential in improving and tailoring psychiatric care to individual needs, the evidence of its clinical effectiveness is still limited. However, we found potential effects on mania symptoms and empowerment. Overall, there were only a few RCTs with formal psychiatric diagnoses to be included in our analyses, and these had moderate risks of bias. Future studies assessing RMBC’s effectiveness and long-term efficacy with larger populations are needed.

## Introduction

Mental health disorders have one of the highest global burdens of disease [1] and are difficult to manage. Hurdles include subjective symptom reporting [2,3], memory biases [4], complex treatment dynamics, and suboptimal coordination during transitions between inpatient and outpatient care settings [5]. Additionally, short and infrequent outpatient appointments contribute to the loss of essential information about symptom progression and treatment side effects [6]. This increases the risk of reduced treatment adherence, worsening conditions, preventable readmissions, and higher health care costs [7,8].

In response to these challenges, there has been a notable increase in the development of diagnostic and therapeutic mobile mental health (MMH) technologies [9-12]. One prominent application of MMH is remote measurement-based care (RMBC), which involves the asynchronous assessment of patient-reported outcomes outside of clinical visits. These assessments can then be used for clinical decision-making and triage purposes [13,14]. Apart from traditional retrospective patient-reported outcome assessment methods, such as validated self-report questionnaires, there is growing interest in ambulatory and diary approaches. These methods, collectively known as ecological momentary assessment (EMA), capture real-time, in situ data on patients’ symptoms and well-being [15]. With advancements in technology, EMA has evolved to allow self-reporting of symptoms via the internet or mobile platforms, including web or online, SMS text messaging, or phone call–based systems [16]. Passive or sensor data integration further enhances the richness of this approach by capturing objective behavioral and physiological indicators in real-world settings, complementing the subjective self-reports provided by patients [13,14]. While RMBC and EMA share similarities in leveraging technology to enhance the understanding and treatment of mental health issues through continuous care, the literature does not always make a clear distinction between the two, often seeing them as part of a continuum in advancing personalized health monitoring and intervention.

Research has consistently demonstrated the benefits of RMBC, in that it may improve clinical outcomes and improve treatment adherence [17-19]. For example, a study involving 6424 participants diagnosed with various psychiatric conditions revealed that providing continuous feedback to therapists on symptom progression was associated with a 2-fold increase in therapeutic effects related to individual functioning, symptom load, interpersonal relationships, and social role performance [20]. Additionally, RMBC has been associated with faster remission rates than standard treatment approaches [21,22] and reduced missed outpatient appointments [21,22]. Moreover, RMBC enables clinicians to make timely and effective adjustments to treatment plans. Patients have reported finding RMBC valuable [23]. RMBC also showed potential to enhance doctor-patient communication and increase treatment motivation [24,25].

Despite the potential benefits, the integration of asynchronous measurement-based care (MBC) using digital solutions remains limited in clinical practice due to time constraints, workflow integration issues, and uncertainties about interpreting and using the data effectively [26]. While MMH technology companies develop extensive solutions, their scientific evaluation often lacks the depth seen in university settings, presenting a significant dissemination barrier for health care providers and insurers. Conversely, the proliferation of MMH technologies has led to numerous pilot and feasibility studies on RMBC systems by clinical research teams, which typically suffer from academic research limitations such as insufficient power and bias reduction strategies, resulting in incoherent and scattered evidence.

Often, the effectiveness of RMBC technologies is difficult to interpret, as they are often integrated into complex intervention bundles with unclear causal pathways and potential confounders. For example, a review found that while feedback from providers improved the therapeutic relationship and promoted help-seeking behavior in young people—both of which may be viewed as proxy markers for improved long-term treatment trajectories—it did not directly impact depression outcomes [27].

Furthermore, considerable variability exists in how data obtained from RMBC are used to inform treatment decisions. Unlike most somatic pharmacotherapy, where objective laboratory measurement results with defined thresholds often lead to discrete, standardized actions (such as medication adjustments), psychiatric treatment often includes a range of potential responses to measured outcomes (primarily based on the subjective reporting) [13]. This induces variance, resulting in heterogeneity in clinical response, further complicating the determination of appropriate end points for evaluating RMBC effectiveness and posing significant challenges for isolating its specific impact within complex, multicomponent interventions.

These unanswered research questions demonstrate the significant need for regular systematic evaluations to identify overarching trends and effects, thereby facilitating the broader adoption of MBC and MMH in routine care.

A 2018 systematic review by Goldberg et al [13] synthesized existing evidence on RMBC, including 36 unique samples, of which 13 were randomized controlled trials (RCTs). While generally supportive of RMBC’s potential, the review highlighted considerable methodological heterogeneity, particularly due to RMBC often being embedded within broader multicomponent interventions [27]. Only 3 studies isolated the effects of RMBC experimentally, with 1 showing greater symptom improvement in the RMBC group and 2 finding no significant differences between intervention and control groups. The feasibility and acceptability of RMBC varied across studies, with promising adherence rates reported but concerns raised regarding decreased responsiveness over time. The review identified the need for more robust evaluations to better understand the isolated clinical impact of RMBC interventions, especially when implemented as part of multicomponent interventions, highlighting the need for further research to clarify its role and potential benefits.

This systematic review and meta-analysis presents a comprehensive overview of current evidence on RMBC in psychiatric care, building upon the findings of Goldberg et al [13]. In contrast to the study by Goldberg et al [13], this study focused on patients who underwent a manualized psychiatric diagnostic assessment and actively engaged with digital tools to report their individual experiences. Specifically, we concentrate on interventions targeting disorder-specific symptoms, relapse reduction, improvement in recovery-oriented outcomes, global functioning, and quality of life. Additionally, we provide a quantitative estimate of effects via a meta-analysis.

## Methods

### Search Strategy and Study Selection

The study adhered to PRISMA-P (Preferred Reporting Items for Systematic Review and Explanation Meta-Analysis Protocols) guidelines (Checklist 1) [28] and was preregistered with the systematic review registry PROSPERO (CRD42022356176). The detailed protocol was published elsewhere [29]. On August 24, 2022, and during the revision on December 21, 2024, we conducted a comprehensive search across 4 online databases (PubMed, Medline, Embase, and PsychINFO) and gray literature using terms related to mental disorders, psychological distress, MBC, and digital technologies (Table S1 in Multimedia Appendix 1).

Inclusion and exclusion criteria were defined by using the PICOS (population, intervention, comparison, outcome, and study) framework (Table S2 in Multimedia Appendix 1) [30]. Studies were included if they (1) targeted adults (≥18 y) diagnosed with a mental health disorder according to the *International Classification of Diseases* (*ICD*) or *Diagnostic and Statistical Manual of Mental Disorders* (*DSM*) [31,32]; (2) implemented interventions centered on the digital assessment of self-reported symptoms or well-being factors to guide clinical decision-making or treatment planning; and (3) reported quantitative outcomes related to symptoms, recovery, functioning, or quality of life. Eligible studies had to be published in English or German. No restrictions were placed on comparator conditions to broaden the evidence base. While the systematic review included randomized and nonrandomized studies, the meta-analysis was restricted to RCTs only.

### Data Extraction

The systematic extraction process was described in the study protocol [29]. Subgroups of authors (T Michnevich and LPSF; FM and LH; JK, CW, and T Muffel) independently reviewed the abstracts and full texts, resolving any discrepancies through group consensus. A comprehensive dataset, encompassing study identification (author, year of publication, DOI, and URL), population (eg, the number of cases and controls, diagnosis, age, gender, and years of preuniversity education), tracking (eg, mode, number and content of items, and frequency), and study characteristics (eg, design, hypotheses, study site, duration, randomization, postassessment period, follow-up, outcomes, and response rate), was extracted. Outcomes were systematically grouped into 6 predefined categories: symptom-focused or disease-specific outcomes, relapse, recovery-focused outcomes (in particular, empowerment), functioning or global functioning, quality of life, and acceptability.

### Data Synthesis and Statistical Analysis

RStudio statistical software (version 2023.09.1+494; Posit PBC) [33] was used for statistical analysis. Demographic variables were descriptively analyzed by calculating means and SDs. A linear regression model was used to explore the impact of daily prompt frequency and the number of tracking items on participant response rate.

### Frequentist Meta-Analysis

#### Data Synthesis

Random-effects meta-analyses were performed using the *metafor* package (version 4.6‐0) [34]. Outcomes were meta-analyzed when at least 3 studies (n>2) reported comparable results. Only instruments with evidence of construct validity or sufficient correlation with other instruments were included. When multiple instruments within a study measured the same construct, the outcome most commonly reported across studies was included to ensure comparability.

We included all measures of psychopathology, even if they were not disease-specific, for example, measures of depression in a sample of patients with psychosis. This approach recognizes the transdiagnostic nature of symptoms and prioritizes symptoms over diagnoses. The full list of constructs and outcomes can be found in Table S3 in Multimedia Appendix 1.

Intention-to-treat data were used for analyses where available. Where outcomes were reported as medians and IQRs, means and SDs were estimated using median-based imputation [35]. If only SEs were reported, SDs were calculated [36]. For trials that reported outcome data at multiple follow-up points, data from the time point immediately after the end of the intervention were used.

Effect sizes for continuous measures were expressed as standardized mean differences (SMDs), calculated by using the pooled SD of the interventions. SMDs are presented as values of Hedges *g*, along with their 95% CI.

#### Assessment of Heterogeneity

Heterogeneity between studies was evaluated using the *I*^2^ statistic and by visual inspection of the forest plots. Heterogeneity was defined as very low, low, medium, and high heterogeneity when *I*^2^ values were <25%, 25% to <50%, 50% to <75%, and ≥75%, respectively [37].

#### Assessment of Publication Bias

Publication bias was evaluated by visual inspection of funnel plots assessing the symmetry of effect size distributions relative to SEs.

### Bayesian Meta-Analysis

To complement the frequentist approach and to better account for uncertainty due to the small number of studies, a random-effects Bayesian meta-analysis was performed using the *bayesmeta* package (version 2.21) [38-41]. Posterior distributions for the overall effect and heterogeneity parameters were estimated via Markov Chain Monte Carlo simulations [42]. Given the paucity of literature on RMBC interventions and the lack of prior knowledge, we used weakly informative priors *µ*=0 and *σ*=4 [43,44]. The prior for between-study heterogeneity *τ*=0.5 was set using a half-normal distribution [45]. Results are presented through marginal posterior density plots, illustrating uncertainty around overall effects and heterogeneity.

### Risk of Bias

The risk of bias was evaluated independently by 2 researchers (FM and T Michnevich), using the Cochrane risk-of-bias tool for randomized trials version 2 (RoB 2) [37]. The researchers assessed potential biases across the 5 domains of the ROB 2 tool: randomization process, effect of assignment to intervention, missing outcome data, measurement of outcome, and selection of reported results [46]. Studies were classified as low risk when all domains were deemed low risk. A study was considered to have some concern if any of the domains raised concerns. The overall risk was designated as high if at least one domain was rated as high risk. Disagreements were resolved through discussion to reach consensus.

## Results

### Selection of Studies

**Figure 1.**
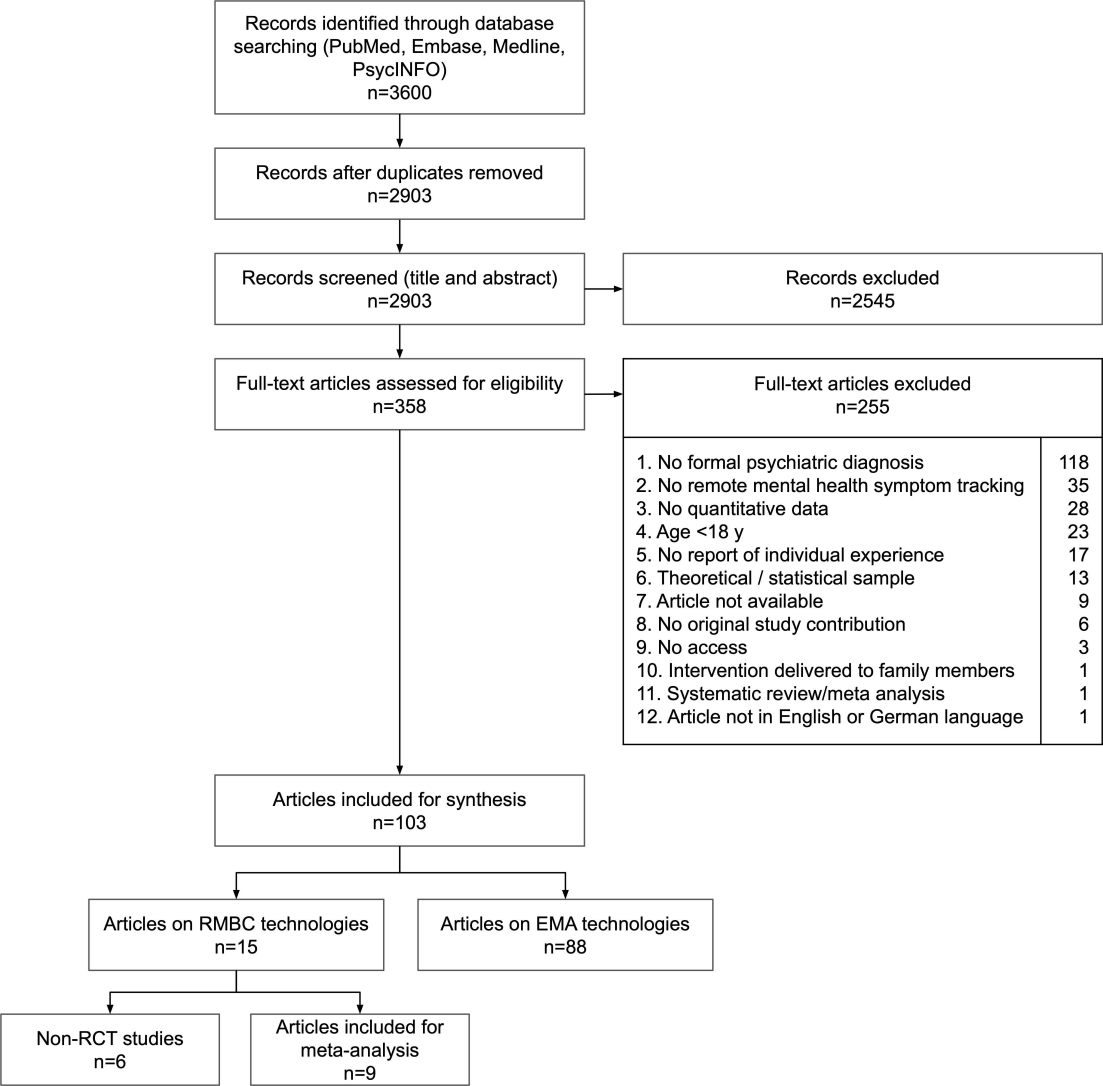
Flowchart of the search and selection process. EMA: ecological momentary assessment; RCT: randomized controlled trial; RMBC: remote measurement-based care.

The database search (N=3599) yielded 2902 records after deduplication (Table S1 in Multimedia Appendix 1), which were screened by title and abstract. Of the 357 records that qualified for full-text analysis, 254 records were excluded for not meeting the inclusion criteria (Figure 1). The most common reason for exclusion (n=118) was the lack of a formal psychiatric diagnosis, using either the *ICD* or *DSM*. The systematic review includes a final sample of 103 studies representing 109 unique samples.

The systematic review revealed that 15 studies examined RMBC sensu stricto (using data to support clinical decision-making or treatment planning), of which the 9 RCT studies were used for meta-analysis. The other studies (n=88) matched the definition of EMA, whereby technologies were also used to collect mental health data remotely in real time, but the data did not have a significant impact on treatment.

### Study Characteristics—Overall Sample

Of the 103 studies that were systematically analyzed, 41 contained healthy or diagnosis-matched control groups. Individual samples (n=109) varied due to overlapping or related datasets (Table S4 in Multimedia Appendix 1). Across the studies, the mean sample size of cases was 80.33 (SD 105.17), with an average participant age of 40.38 (SD 7.70) years and a mean study-level proportion of 56.79% (SD 22.43) female participants. For the studies including control groups, the average sample size was 62.49 (SD 71.70) controls with an average age of 41.56 (SD 6.92) years and a mean proportion of 55.04% (SD 20.42) female participants. Educational attainment was reported through various metrics, the most frequent being total years of education, which averaged at 13.62 (SD 1.22) for cases and 13.52 (SD 1.25) for control samples. The most common population was participants with schizophreniform disorders (n=21, 20.4%; ie, F2x diagnoses), followed by bipolar disorder (n=19, 18.4%). Most studies (n=70) used digital prompts formulated by the study team, while 15 studies used validated questionnaires. Six studies used a combination of both methods, and 7 studies incorporated prompts that were individually generated by the participants themselves. The predominant mode of remote data collection involved smartphones or mobile phones owned by the participants themselves. Adherence to data entries was mainly measured by the percentage of total measurements entered by the participants.

### Study Characteristics—RMBC Studies

Table 1 and Tables S5 and S6 in Multimedia Appendix 1 report information extracted from the RMBC studies. Chermahini et al [47] reported demographic information jointly for the case and control groups, which is why there are only 8 unique samples for the demographic data, not 9. Across the 8 samples, the intervention groups had a mean of 66 (SD 58.98) cases, with a mean age of 38.53 (SD 7.14) years and a mean proportion of 54.75% (SD 17.93) female participants across studies. Comparably, the control groups (n=8) had a mean of 58.63 (SD 60.38) participants, with a mean age of 39.93 (SD 7.08) years and a mean proportion of 52.89% (SD 15.25) female participants across studies. Most of the studies (n=6) did not report on education, and the remaining studies used varying measures. Three studies included patients with schizophreniform disorders [48-50]; others included patients with bipolar disorder (n=2), borderline personality disorder (n=1), generalized anxiety disorder (n=1), and a range of different diagnoses or transdiagnostic symptoms (n=2). On the patient side, the majority of RMBC systems (n=6) were mobile phone- or smartphone-based. All interventions consisted of self-administered symptom tracking along with additional formalized (eg, psychotherapy) or informal psychiatric or psychotherapeutic support.

**Table 1.** Characteristics of remote measurement-based care (RMBC) studies included in the meta-analysis.

Study	Participants, n (%)		Psychiatric disorder	Setting	Treatment	Control	RMBC device	Isolated RMBC	Response rate (%)	ITT^a^	Clinical effectiveness or efficacy
	Treatment group	Control group									
Cullen et al (2020) [48]	28 (68.3)	13 (31.7)	Schizophrenia and schizoaffective disorder	Hospital-based community psychiatry program	Self-administered assessment and automated intervention with additional support by health care providers	TAU^b^	Mobile phone	Yes	N/A^c^	No	Positive
Chermahini et al (2024) [47]	Group 1: 45 (46.9) Group 2: 51 (53.1)	N/A	Generalized anxiety disorder	Psychiatric outpatient clinics	Treatment 1: electronic cognitive behavioral therapy including homework with personalized feedback from health care providers	Treatment 2: weekly asynchronous online mental health check-in questions with personalized clinical feedback	Online platform (treatment 1), asynchronous messaging system (online psychotherapy tool; treatment 2)	Yes	N/A	Yes	Positive
Ebert et al (2013) [51]	21 (100)	N/A	Affective disorders Neurotic, stress-related, and somatoform disorders Behavioral syndromes associated with physiological disturbances and physical factors Disorders of adult personality and behavior	Psychiatric inpatient and outpatient treatment	In-person psychotherapy Internet-based maintenance therapy with additional coaching support	In-person psychotherapy	Not specified (web-based)	No	N/A	Yes	Positive
Faurholt-Jepsen et al (2020) [49]	85 (65.9)	44 (34.1)	Bipolar disorder	Psychiatric outpatient clinics	Self-administered symptom assessment with additional support from health care professionals	TAU	Smartphone	No	72.6	Yes	No significant difference
Faurholt-Jepsen et al (2021) [50]	47 (48.0)	51 (52.0)	Bipolar disorder	Psychiatric outpatient clinics	Self-administered symptom assessment with additional support from health care professionals	TAU	Smartphone	No	80.6	Yes	No significant difference
Gallinat et al (2021) [52]	12 (48.0)	13 (52.0)	Schizophrenia spectrum disorder	Research clinic	Collaborative care Self-administered symptom assessment with additional support from health care professionals	TAU	Smartphone or mobile phone	No	70.7	N/A	Positive
Laursen et al (2021) [36]	42 (53.8)	36 (46.2)	Borderline personality disorder	Psychiatric outpatient clinics	In-person psychotherapy (DBT^d^); collaborative care Self-administered symptom assessment and psychoeducation	In-person psychotherapy (DBT) Paper-based diary cards	Smartphone	No	N/A	Yes	Positive and negative
Lewis et al (2020) [53]	40 (49.4)	41 (50.6)	Schizophrenia spectrum disorder	Outpatient mental health facility	Self-administered symptom assessment with additional support from health care professionals	TAU	Smartphone	No	N/A	Yes	Positive
Spaniel et al (2015) [54]	74 (50.7)	72 (49.3)	Schizophrenia and schizoaffective disorder	Psychiatric outpatient clinic	Self-administered symptom assessment with additional support from psychiatrists	TAU	Mobile phone to computer SMS interface	No	N/A	Yes	No significant difference

a ITT: intention to treat.

b TAU: treatment as usual.

c N/A: not applicable.

d DBT: dialectical behavior therapy.

### Effectiveness of RMBC Interventions

While most studies found RMBC interventions to be effective, the others found no effects (n=3) or mixed results. Faurholt-Jepsen et al [49] found no benefit of a 9-month self-administered symptom assessment that provided patients with automated predictions of future mood states. In an exploratory subgroup analysis, patients in the intervention group were more likely to experience a relapse of depressive symptoms than patients receiving usual outpatient care.

### Adverse Effects of RMBC

Three RMBC studies reported adverse or potential negative effects of the interventions. These included technical malfunctioning, psychological distress attributed to prompts [53], hospitalization within the trial period (notably considered an outcome parameter, not an adverse effect, by several other studies) [54]; and changes to patient-therapist interactions due to the new technology [36].

### Frequentist Random Effects Meta-Analysis

Data related to relapse and readmission rates were inconsistent between the studies with differing periods of observation. Thus, no meta-analysis of the data was possible.

### Meta-Analysis of Symptom-Focused Outcomes

**Figure 2.**
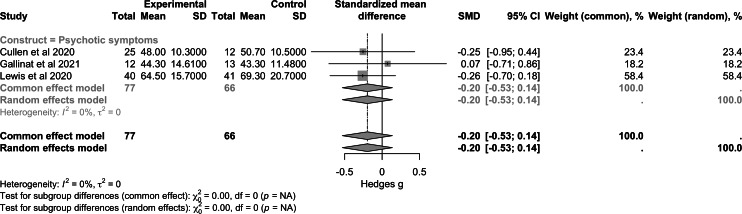
Forest plot of pooled effect on psychotic symptoms [48,52,53].

**Figure 3.**
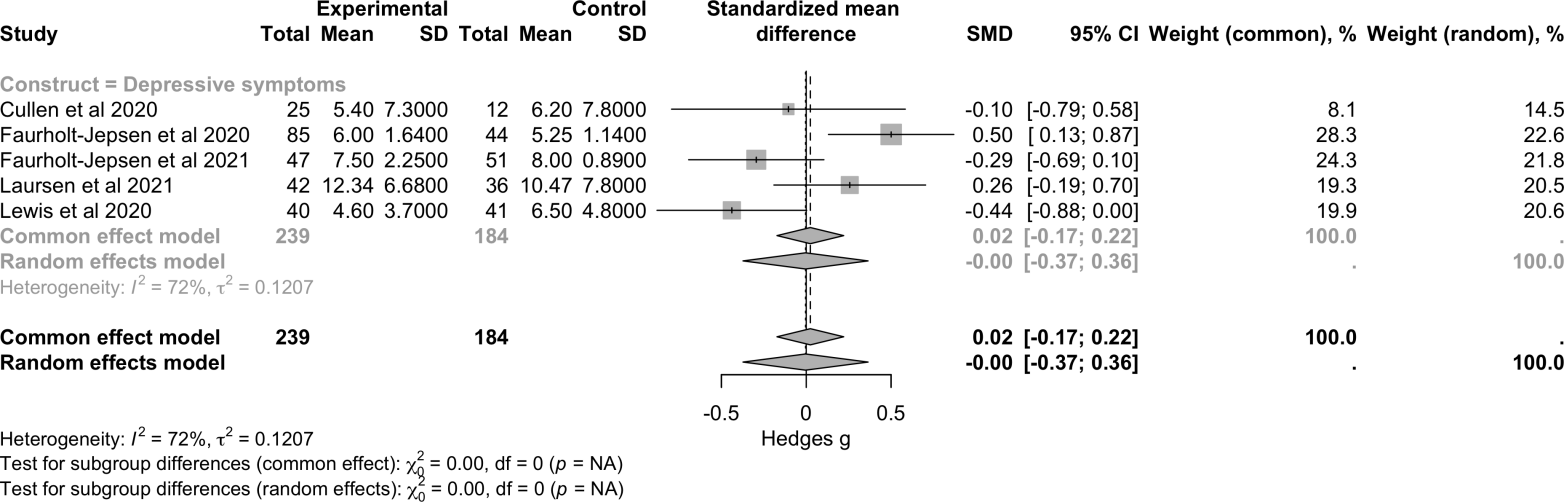
Forest plot of pooled effect on depressive symptoms [36,48-50,53].

**Figure 4.**
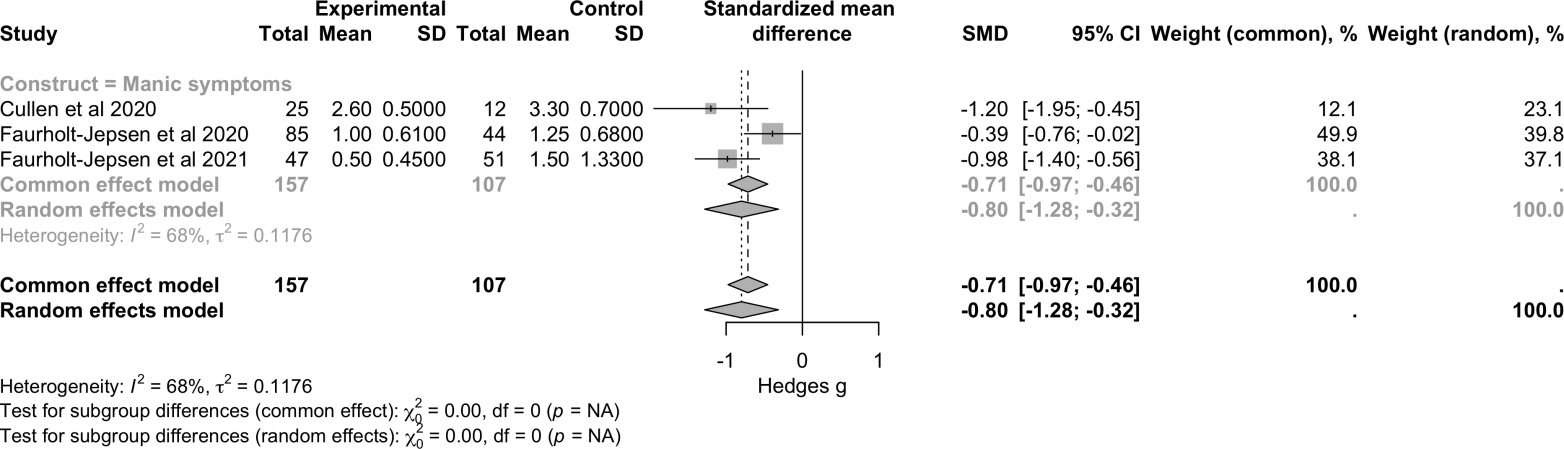
Forest plot of pooled effect on manic symptoms [48-50].

**Figure 5.**
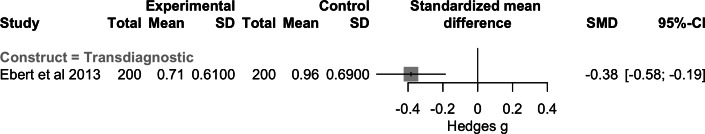
Forest plot of effect on transdiagnostic symptoms [51].

Regarding psychotic symptoms (Figure 2), data from 3 studies (n=143) showed a small nonsignificant effect (SMD −0.20, 95% CI −0.53 to 0.14; *P*=.20). For depressive symptoms (Figure 3), a larger sample of 5 trials (n=423) showed a nonsignificant overall effect (SMD −0.00, 95% CI −0.37 to 0.36; *P*>.99). For manic symptoms (Figure 4), data from 4 studies (n=264) revealed a moderate to large significant effect of RMBC interventions (SMD −0.80, 95% CI −1.28 to −0.32; *P*<.001). Data from one large transdiagnostic study (Figure 5; n=400) suggested a moderate and significant effect size with an SMD of −0.29 (95% CI −0.40 to −0.17; *P*<.001). Between-study heterogeneity was low for psychotic symptoms (*I*^2^=0%), moderate for depressive symptoms (*I*^2^=72%), and moderate for manic symptoms (*I*^2^=68%), suggesting varying degrees of similarity between the studies within each construct.

### Meta-Analysis of Empowerment, Quality of Life, and Functioning

**Figure 6.**
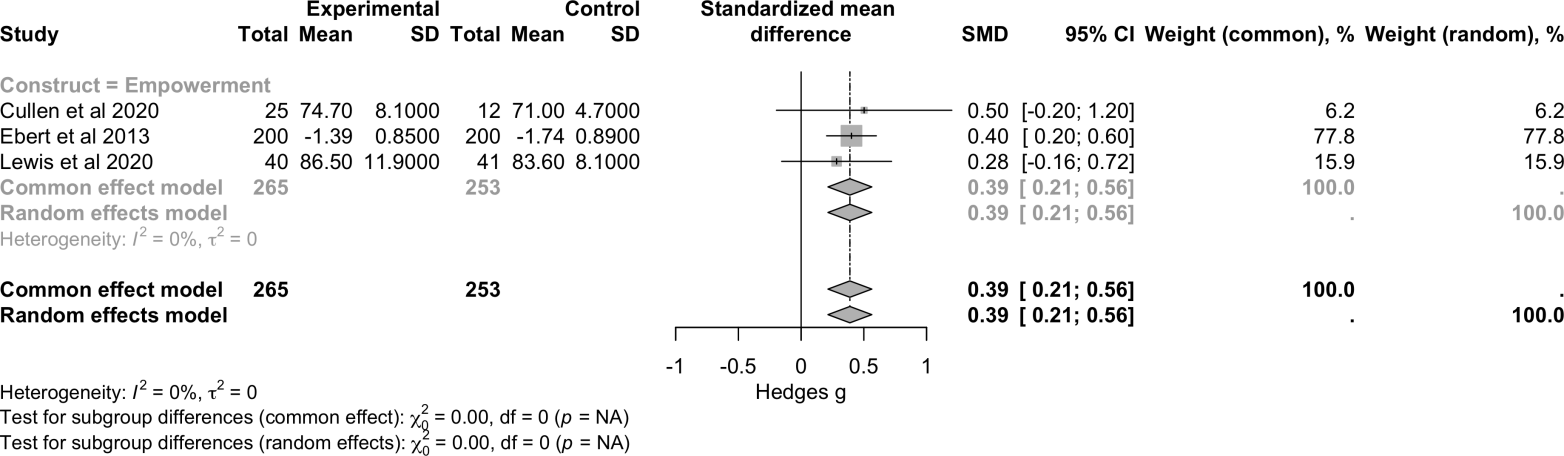
Forest plot of pooled effect on empowerment and self-efficacy [48,51,53].

**Figure 7.**
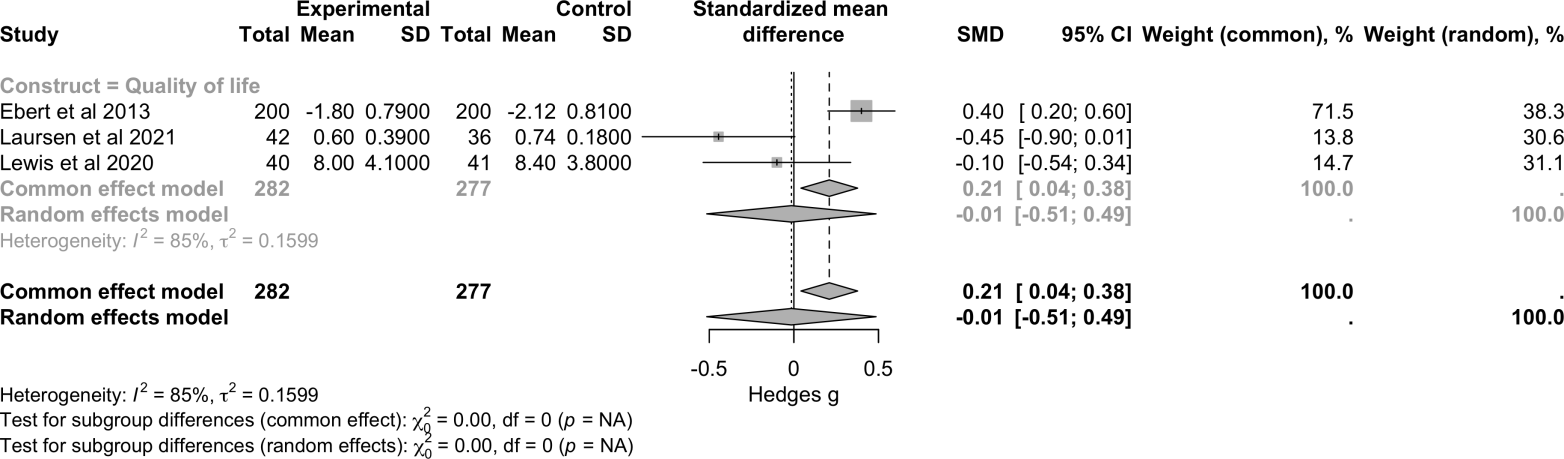
Forest plot of pooled effect on quality of life [36,51,53].

**Figure 8.**
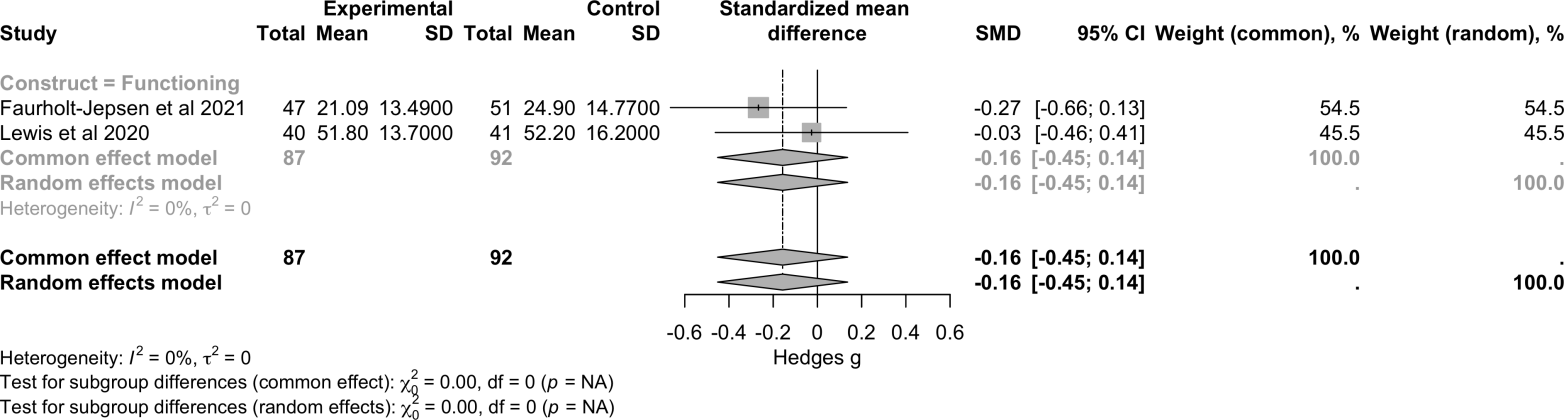
Forest plot of pooled effect on functioning [50,53].

For the construct of empowerment and self-efficacy (Figure 6), pooled data from 3 studies (n=518) demonstrated a small-to-moderate positive effect (SMD 0.39, 95% CI 0.21 to 0.56; *P*<.001). Regarding quality of life (Figure 7), combined results from 4 studies (n=601) showed a nonsignificant effect (SMD −0.01, 95% CI −0.40 to 0.38; *P*>.99). For functioning (Figure 8), the analysis included 2 studies (n=179) and reported a nonsignificant effect (SMD −0.16, 95% CI −0.45 to 0.14; *P*>.99). Heterogeneity between the studies was zero for empowerment and functioning (*I*^2^=0%) and high for quality of life (*I*²=85%).

### Bayesian Meta-Analysis

In general, the Bayesian meta-analysis yielded similar results to the frequentist meta-analysis, but there was only one significant result: the weighted pooled effect size with a mean estimate of −0.79 (95% CI −1.44 to −0.20), for the reduction in manic symptoms associated with RMBC interventions. There was moderate heterogeneity *τ*=0.36 (0.00-0.84). The prediction interval of −1.99 to 0.34 reflected moderate uncertainty in predicting new effects based on current data (Figure S7C in Multimedia Appendix 1). The effect on empowerment that was significant in the frequentist analysis showed an effect size of 0.39 and the CI crossed zero (95% CI −0.02 to 0.79). Other analyses showed nonsignificant effects on the outcomes assessed (Figures S7A,B,D-F and S8 in Multimedia Appendix 1).

### Risk of Bias

**Figure 9.**
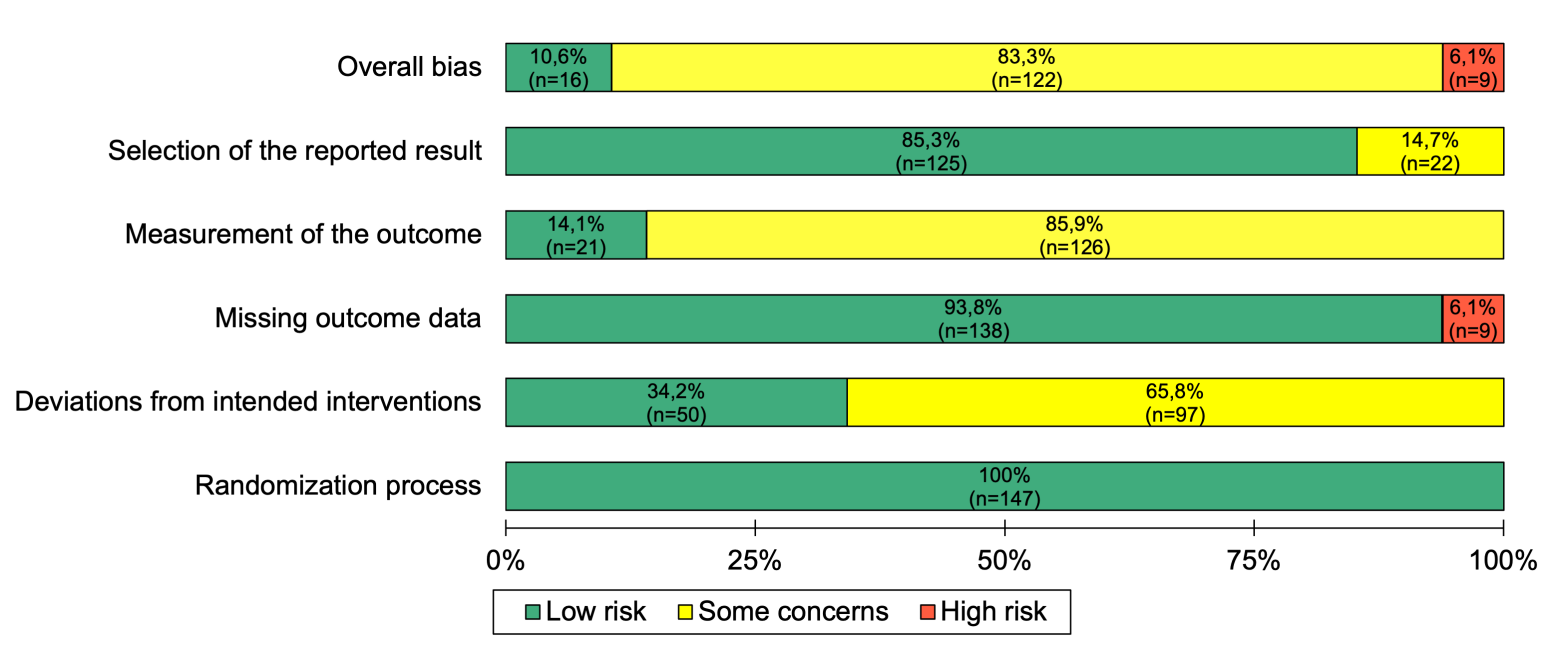
Cochrane risk of bias summary. Authors’ judgments about each risk of bias item across all assessment time points.

The overall risk of bias indicates that the majority of outcomes were of concern to the reviewers (Figure 9). This was largely due to participants, caregivers, or assessors having been aware of the assigned digital interventions, which made it difficult to assess the outcomes, particularly since many relied on participant-reported data. Further, the effect of assignment to the intervention raised concerns about deviations from the intended interventions. Specifically, the outcomes of the Boston University Empowerment Scale in Cullen et al [48] were rated as having a high risk of bias because missing data were replaced by scale means from follow-up data. In addition, the reviewers identified a high risk of bias in the outcome data from Chermahini et al [47] due to high dropout rates and missing data. A full assessment of each outcome is provided in Figure S10 in Multimedia Appendix 1.

### Tracking and Adherence—Overall Sample

Typically, participants were prompted to complete questionnaires >1× per day, followed by daily EMA, with the number of items ranging between 1 and 43 (mean 13.87, SD 10.55) per session (in 16 studies, the number of items was unclear, and in 5 studies, the number of items varied). The most granular tracking data were collected by Freedman et al [55] with 128 to 136 tracking items per day, amounting to a minimum of 896 individual data points per week. Thirteen studies included additional passive data sensing such as GPS, phone usage, speech activity, ambient noise and light, and sleep activity. Forty-seven studies provided a metric of EMA or RMBC adherence with an overall mean response rate of 74.64% (SD 13.04%) to the prompts.

### Association Between Tracking Frequency and Adherence

**Figure 10.**
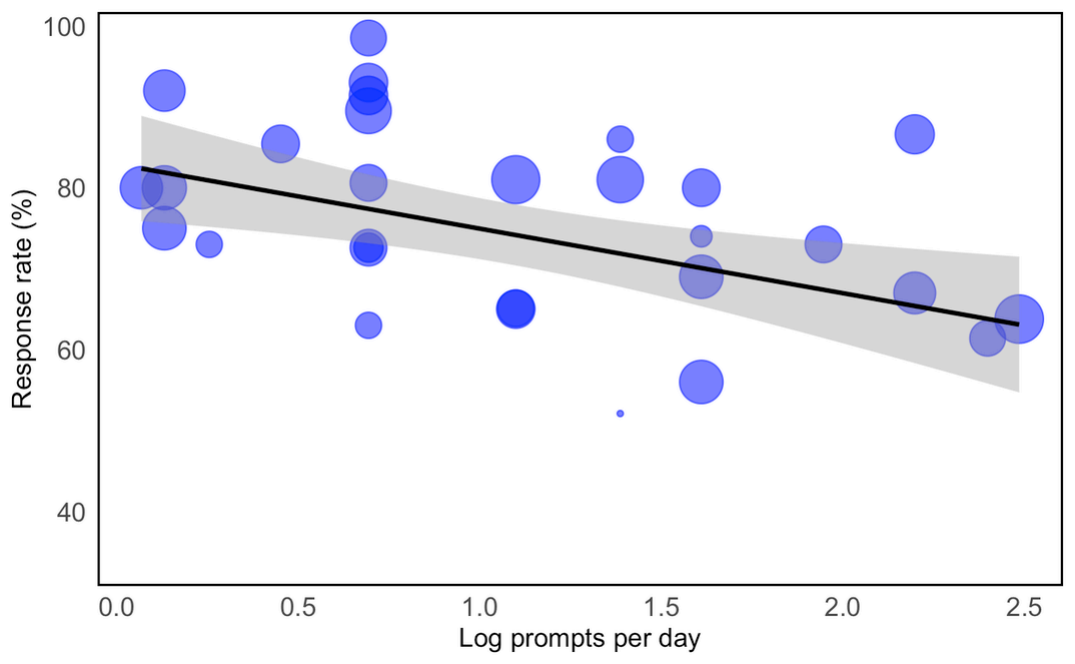
Bubble plot visualization with the predicted probability line with 95% CI (gray area) for the response rate (%) as a function of the log-transformed number of daily prompts on the x-axis (est=−7.121, *t_26_*=−2.540; *P*=.02) and the number of tracking items per prompt, represented as the bubble size (est=3.379, *t_26_*=1.306; *P*=.20).

A linear regression model investigated the effect of the number of prompts per day and the number of tracking items per day on response rate (Figure 10). The model results (Table 2) showed a significant negative effect of the logarithm of the number of prompts per day (*P*=.02) and no effect of the number of tracking items on the response rate. Diagnostic plots showed no obvious violations of the key assumptions (Table S9 in Multimedia Appendix 1). There was no multicollinearity between the independent variables as all variance inflation factor values were less than 5. The model’s overall fit was sufficiently good, given the residual plots (adjusted *R*^2^=0.0173), but other variables may have affected the response rate. Due to omitted variable bias, the model may overestimate the effect of predictors.

**Table 2.** Summary table of the linear regression model results^a^.

Coefficients	Estimate	SE	*t* test (*df*)	*P* value	VIF^b^
(Intercept)	75.617	7.217	1,047,810.072 (26)	<.001	—^c^
Log prompts per day	−7.121	—	−2.540 (26)	.02^d^	1.04
Log tracking items	3.379	2.587	1.306 (26)	.20	1.04

a Linear model: response rate approximately log prompts per day + log number of tracking items per prompt.

b VIF: variance inflation factor.

c not applicable.

d *P*<.05.

## Discussion

### Principal Results

Given the widespread access to smartphone technology, the steadily advancing EMA and RMBC research, and the limited evidence through RCTs and systematic studies on interventions in mental health care, we aimed to review and evaluate the diverse literature in the field. This systematic review targeted the study design features and procedures of EMA and RMBC across psychiatric disorders. Concurrently, the meta-analysis aggregated and examined the effects of RCTs implementing RMBC interventions, focusing on outcomes pertinent to clinical efficacy and recovery-oriented outcomes.

Overall, we found compliance and retention rates for RMBC and EMA technologies to be encouraging, aligning with previous findings in broad EMA research [56,57]. We found that more prompts, but not tracking items, negatively affected the response rate. This observation corroborates the meta-analytic evidence by Vachon et al [57], who noted a positive correlation between compliance and fewer daily prompts as well as longer intervals between prompts in severe mental illnesses [50]. It also confirms findings from a systematic review by Williams et al [58], showing that higher numbers of tracking items per prompt were not associated with reduced compliance in clinical samples; in healthy individuals, however, more items were indeed associated with lower compliance. Up to 5 random EMA prompts per day have been deemed optimal for longitudinal studies [59]. However, in the context of substance use disorders, Jones et al [60] reported that compliance was not significantly impacted by the number of prompts per day or the duration of the assessment period. Our results support the evidence for severe mental illness and are in favor of longer intervals between successive evaluations to maximize, potentially influenced by the low representation of substance use disorders within our sample due to a lack of formal diagnosis.

### Methodology

During the systematic review of the literature, a prominent distinction was identified between RMBC and EMA. Despite their shared aim of collecting subjective, real-time data from remote settings, they serve distinct purposes from a clinical perspective. While RMBC encompasses elements of EMA, it is directed toward informing health care decisions and interventions, for example, supporting real-time and asynchronous treatment adjustments or scheduling of visits [13,14]. For patients, both EMA and RMBC interfaces facilitate reflection on symptoms, with many offering data summaries on symptom trajectories. As the primary difference between RMBC and EMA, RMBC focuses on enabling clinicians to formulate recommendations and implement treatment adjustments based on real-time data. As a result, patients may perceive RMBC as involving closer monitoring, which in turn is subject to individual preference. Some patients may interpret RMBC as an invasion of their autonomy and privacy, while others may find comfort in the increased level of monitoring, viewing it as an additional safety measure.

In our meta-analysis of RMBC interventions, we investigated the transdiagnostic benefits of the technologies often emphasized in the literature. Therefore, the analysis considered psychopathological, cross-diagnostic constructs rather than individual diagnostic groups of participants. In addition to the well-known challenges of different design features and procedures when integrating and aggregating data from EMA and RMBC studies [13,56], this aspect may have increased heterogeneity. Overall, we did not observe clear effects of RMBC interventions for most of the constructs we analyzed, that is, depressive, psychotic symptoms, quality of life, and daily functioning. This is consistent with the results of Goldberg et al [13], who assumed a general effect of RMBC interventions but did not draw any conclusions about specific effects due to the small number of RCTs.

There was no overlap between the studies we included in our review and those by Goldberg et al [13]. This is because we only included studies with manualized psychiatric diagnostic procedures and applied a narrower definition of RMBC, emphasizing a possible clinical need including self-monitoring for decision-making and therapy planning. Goldberg et al [13] did not set a formal psychiatric diagnosis as an inclusion criterion and used a wider definition of RMBC, in particular regarding its direct effects on treatment trajectories.

Both frequentist and Bayesian meta-analyses demonstrated a significant effect on the reduction of manic symptoms when pooling data from 3 studies [48-50]. From both RCTs by Faurholt-Jepsen et al [49,50], which found no significant effect on emotional (depressive and manic) symptoms, medians and SEs were converted to means and SDs. It is generally known that the standardization of results can introduce flaws in meta-analysis [61]. Therefore, this result has to be considered with caution. Although there is scarce systematic evidence on the effects of RMBC on manic or hypomanic symptoms, there appears to be a benefit from clinical practice due to the dynamic and fluctuating nature of the symptoms and also clear recommendation on symptom tracking in guidelines [62,63]. The exploratory analysis by Faurholt-Jepsen et al [49] underlined that smartphone-based monitoring may reduce the risk of relapse of manic episodes but increase the risk of relapse of depressive episodes. This finding is underscored by a systematic review by Hennemann et al [64], who examined internet- and mobile-based tools for psychiatric aftercare and relapse prevention. They found small to moderate symptom reduction, with the best evidence for depression and anxiety [64].

In the frequentist meta-analysis, we found an effect of RMBC interventions on empowerment and self-efficacy. Of note, the largest study (n=200) by Ebert et al [51] contributed the largest weight to this result. Although validated, the instrument used includes a limited set of 5 items as a subscale of the HEALTH-49 questionnaire and has no proven correlation with the BUES [65]. These results should also be evaluated with caution in light of the results of the Bayesian meta-analysis where the effect was also detectable but the CI includes zero. Overall, self-efficacy seems a promising target for RMBC tools.

### Quality of Evidence

A keyword search of the manuscripts in this systematic review and meta-analysis showed that unfortunately, none of the publications mentions the CONSORT (Consolidated Standards of Reporting Trials) checklist or its EHEALTH (Electronic Health) extension. Additionally, some essential outcome measures were missing from the vast majority of studies.

For one, adverse events were reported by only a fraction of studies. This is particularly surprising as adverse events pertaining to the technologies and treatment modalities may be easily transferable, constituting an efficient knowledge transfer and reducing potential harm to study and clinical populations. Direct adverse psychological effects of symptom tracking include anxiety or obsessiveness about choosing the “wrong” answer and an increased awareness of symptoms mentioned in questions or prompts [60], which may increase disease burden. Symptom tracking has also been found to potentially amplify symptoms or create the illusion of symptom amplification for patients and clinicians through over-reporting [66].

Indirect negative effects include feelings of guilt when tracking is missed, cognitive dissonance due to continuous confrontation with mental illness, and boredom or fatigue [67]. Symptom-tracking apps may also promote individualist models of illness that negate social determinants of health and make patients indirectly responsible for their illness if they refuse or fail to track symptoms [68]. Shared decision-making and using routine outcome monitoring collaboratively could address this concern and has been shown to increase the working alliance in mental health care [66].

Incomplete or absent adverse event reporting may be linked to the circumstance that many EMA or RMBC studies are financed by industry, and funding for further product development may be dependent upon its evaluation, constituting a potential conflict of interest. This conflict of interest has to be taken into account when evaluating the effectiveness of MMH apps.

A further concern identified within the analyzed studies is the limited reporting of adherence and response rates. This underreporting poses significant methodological and interpretive challenges. Adherence and response rates are critical indicators of the feasibility and acceptability of interventions among participants. Limited or absent reporting of these metrics hinders a full understanding of intervention effectiveness and the factors influencing user engagement. Without clear insights into adherence and response rates, it becomes difficult to determine the reliability and generalizability of study findings. A further obstacle here is the multitude of metrics used, for example, the percentage of total assessments completed [69], the percentage of days within the observation period on which assessments were completed [70], or a binary definition of compliance or noncompliance based on a cutoff of completed assessments [71]. We therefore strongly emphasize the importance of standardized adherence reporting. Minimally, authors should report the total share of assessments completed within the study population, the average percent of assessments completed per person, and factors associated with nonadherence (ie, demographics or time-varying factors [72,73]).

A common barrier to evidence synthesis that affected this research is the heterogeneity of study populations, specifically the lack of a formal psychiatric diagnosis in many study samples. About a third of the full-text articles were primarily excluded for this reason. As many of the excluded study populations most likely fulfilled *DSM-5* or *ICD-10* diagnostic criteria, this issue underscores the importance of standardized diagnostic criteria and rigorous documentation of participant characteristics in clinical research.

### Strengths and Limitations

On the one hand, our inclusion criteria required a formal psychiatric diagnosis, which led to the exclusion of many studies that used EMA and RMBC technology. On the other hand, this criterion also strengthens the methodology by ensuring a higher standard of diagnostic rigor within the included studies. In addition, our focus on RCTs increases the reliability of our findings by selecting evidence from studies with robust experimental designs. The heterogeneity of terminology in studies exploring similar concepts, such as EMA or RMBC, may have led to the inadvertent omission of relevant research. To meta-analyze constructs, outcome constructs were pooled, reducing their discriminatory power, possibly leading to an underestimation or nondetection of effects [74]. Even after an extended screening period during the revision phase, only 16 studies met our inclusion criteria and were included in the analysis, highlighting a gap between progressive technological innovation and rigorous clinical validation.

### Recommendations

From studying the existing evidence on EMA and RMBC for mental health care, we recommend adherence to standardized reporting guidelines such as CONSORT-EHEALTH (Consolidated Standards of Reporting Trials of Electronic and Mobile Health Applications and Online Telehealth). To effectively analyze acceptability and adherence, we suggest the establishment of standards for response-rate measurement. To gain feedback on the user experience as well as the perspective of health care providers with RMBC products, mixed methods designs can provide valuable insights for challenges in implementations of such measures.

### Conclusions

In conclusion, our systematic review and meta-analysis underscore the potential of RMBC interventions in enhancing the management of mental health conditions, particularly in reducing symptom severity in mania and increasing empowerment. While demonstrating promising effects on adherence and symptom-specific outcomes, the variability in intervention effectiveness and concerns about bias highlight the need for further research and refinement to optimize the implementation of RMBC within mental health care systems.

## Supplementary material

10.2196/63088 Multimedia Appendix 1 Supplementary materials for the systematic review and meta-analysis, including search strategies, eligibility criteria, outcome mappings, study and intervention characteristics, forest plots, risk of bias assessments, and R code.

10.2196/63088 Checklist 1 PRISMA-P (Preferred Reporting Items for Systematic Review and Explanation Meta-Analysis Protocols) Checklist.

## Abbreviations

CONSORT: Consolidated Standards of Reporting Trials
CONSORT-EHEALTH: Consolidated Standards of Reporting Trials of Electronic and Mobile Health Applications and Online Telehealth
CONSORT-MHEALTH: Consolidated Standards of Reporting Trials-Mobile Health
*DSM*: *Diagnostic and Statistical Manual of Mental Disorders*
EMA: ecological momentary assessment
*ICD*: *International Classification of Diseases*
MBC: measurement-based care
MMH: mobile mental health
PICOS: population, intervention, comparison, outcome, and study
PRISMA-P: Preferred Reporting Items for Systematic Review and Explanation Meta-Analysis Protocols
RCT: randomized controlled trial
RMBC: remote measurement-based care
RoB-2: Cochrane risk-of-bias tool for randomized trials version 2
SMD: standardized mean difference
