# Supplementary material for: Remote Measurement-Based Care Interventions for Mental Health: Systematic Review and Meta-Analysis
Source: JMIR Ment Health. 2026 Jan 28;13:e63088. doi: 10.2196/63088 (PMC12849610; doi:10.2196/63088)
Supplement: Multimedia Appendix 1 [file mental-v13-e63088-s001.docx]

**Multimedia Appendix**

**S1.** Search strategies, search dates, number of results retrieved per database, and number of references included for review.

| **Database** | **Date of search** | **Results** | **Search syntax** |
| --- | --- | --- | --- |
| PubMed | 24.08.2022 | 1456 | ("Mental Disorders" [MeSH Terms] OR "Stress, Psychological" [MeSH Terms]) AND ("Self Report" [MeSH Terms] OR "patient reported outcome measures" [MeSH Terms] OR "ecological momentary assessment" [MeSH Terms] OR ("symptom*"[All Fields] AND "monitor*"[All Fields]) OR ("symptom*"[All Fields] AND "assessment*"[All Fields]) OR ("patient"[All Fields] AND "monitor*"[All Fields]) OR ("remote*"[All Fields] AND "monitor*"[All Fields]) OR ("remote*"[All Fields] AND "assessment*"[All Fields]) OR ("remote*"[All Fields] AND "symptom*" [All Fields] AND "monitor*"[All Fields]) OR ("remote*"[All Fields] AND "symptom*" [All Fields] AND "assessment*"[All Fields]) OR ("remote*"[All Fields] AND "patient"[All Fields] AND "monitor*"[All Fields]) OR ("measurement*"[All Fields] AND "based"[All Fields] AND "care"[All Fields]) OR ("ambulator*"[All Fields] AND "assessment*"[All Fields]) OR ("electronic*"[All Fields] AND "diar*"[All Fields]) OR ("personal*" [All Fields] AND "diar*"[All Fields])) AND ("digital technology"[MeSH Terms] OR ("mobile"[All Fields] AND "application*"[All Fields]) OR "telemedicine"[MeSH Terms] OR "smartphone"[MeSH Terms] OR "cell phone"[MeSH Terms] OR "text messaging"[MeSH Terms] OR "electronic mail"[MeSH Terms] OR "internet based intervention"[MeSH Terms] OR "sms"[All Fields] OR "short message service" [All Fields] OR ("e-mental"[All Fields] AND "health"[MeSH Terms]) OR ("digital*"[All Fields] AND "mental health"[MeSH Terms]) OR ("technology"[MeSH Terms] AND "based"[All Fields] AND "intervention*"[All Fields])) |
|  | 21.12.2024 | 154 |  |
| Medline / Embase | 24.08.2022 | 919 | 1. Mental Disorders/  2. Stress, Psychological/  3. 1 or 2  4. Self Report/  5. Patient Reported Outcome Measures/  6. Ecological Momentary Assessment/  7. (symptom* and monitor*).mp.  8. (symptom* and assessment*).mp.  9. (patient* and monitor*).mp.  10. (remote* and monitor*).mp.  11. (remote* and assessment*).mp.  12. (remote* and symptom* and monitor*).mp.  13. (remote* and symptom* and assessment*).mp.  14. (remote* and patient* and monitor*).mp.  15. (measurement* and based and care).mp.  16. (ambulator* and assessment*).mp.  17. (electronic* and diar*).mp.  18. (personal* and diar*).mp.  19. 4 or 5 or 6 or 7 or 8 or 9 or 10 or 11 or 12 or 13 or 14 or 15 or 16 or 17 or 18  20. Digital Technology/  21. (mobile and application*).mp.  22. Telemedicine/  23. Smartphone/  24. Cell Phone/  25. Text Messaging/  26. Electronic Mail/  27. Internet-Based Intervention/  28. sms.mp.  29. (short and message and service).mp.  30. (e-mental and health).mp.  31. (digital and mental and health).mp.  32. (technology and based and intervention*).mp.  33. 20 or 21 or 22 or 23 or 24 or 25 or 26 or 27 or 28 or 29 or 30 or 31 or 32  34. 3 and 19 and 33 |
|  | 24.12.2024 | 418 |  |
| PsycINFO | 24.08.2022 | 523 | (Mental Disorder* OR psychological stress) AND (Self Report OR patient reported outcome measures OR ecological momentary assessment OR (symptom* AND monitor*) OR (symptom* AND assessment*) OR (patient AND monitor*) OR (remote* AND monitor*) OR (remote* AND assessment*) OR (remote* AND symptom* AND monitor*) OR (remote* AND symptom* AND assessment*) OR (remote* AND patient AND monitor*) OR (measurement* AND based AND care) OR (ambulator* AND assessment*) OR (electronic* AND diar*) OR (personal* AND diar*))  AND (digital technology OR (mobile AND application*) OR telemedicine OR smartphone OR cell phone OR text messaging OR electronic mail OR internet based intervention* OR sms OR short message service" OR (e-mental AND health) OR (digital* AND mental health) OR (technology AND based AND intervention*)) |
|  | 24.12.2024 | 129 |  |
| **Total references** | | 3599 |  |
| **References for review** | | 2902 | After the removal of duplicates |

**S2.** Inclusion and exclusion criteria related to the PICOS design

| **PICOS domain** | **Inclusion criteria** | **Exclusion criteria** |
| --- | --- | --- |
| P | Participants were diagnosed with a mental health disorder defined by DSM-V or ICD-10 F-diagnoses or Z73 (Problems related to life-management difficulty) | No formal psychiatric diagnosis |
|  | Adults |  |
|  |  | Intervention delivered to family members (either as the target recipients of the intervention or in addition to the patients) |
| I | Remote mental health symptom tracking | No remote mental health symptom tracking (including psychiatric symptom only tracking immediately before, after, or during a clinical encounter) |
|  | Report of self-reported (individual) experience | No report of individual experience (including psychiatric symptom tracking only through passive sensing / monitoring) |
| C | Not specified | Not specified |
| O | Quantitative data |  |
|  | Symptom-focused outcomes |  |
|  | Relapse |  |
|  | Recovery-focused outcomes (e.g. empowerment, self-efficacy, hope, social connectedness) |  |
|  | (Global) functioning |  |
|  | Quality of life |  |
| S | Longitudinal observational study designs, including retrospective and prospective studies |  |
|  | Experimental study designs, including randomised controlled trials and cluster randomised controlled trials | Theoretical or statistical models |
|  | Mixed methods study designs | Systematic reviews and meta-analyses |
|  | Feasibility or pilot study designs | Case studies |
|  | Manuscript written in English or German | No original research contribution (letters to the editors, opinions) |

**S3.** Mapping of individual instruments to overarching outcome categories

| **Outcome** |  | **Instrument** | **Study** |
| --- | --- | --- | --- |
| Symptom-focused or disease-specific | Psychosis | 30-item Positive and Negative Syndrome Scale (PANSS) | Cullen et al. 2020; Gallinat et al. 2021; Lewis et al. 2020 |
|  | Depression | 10- item Montgomery-Asberg Depression Rating Scale (MADRS) | Cullen et al. 2020 |
|  |  | 17-item Hamilton Depression Rating Scale (HDRS) | Faurholt-Jepsen et al. 2019; Faurholt-Jepsen et al. 2020 |
|  |  | 9-item Patient Health Questionnaire (PHQ-9) | Laursen et al. 2021 |
|  |  | 9-item Calgary Depression Scale for Schizophrenia (CDSS) | Lewis et al. 2020 |
|  | Mania | 11-item Young Mania Rating Scale (YMRS) | Cullen et al. 2020; Faurholt-Jepsen et al. 2019; Faurholt-Jepsen et al. 2020 |
|  | Suicide | 4-item Suicidal Behaviors Questionnaire-Revised (SBQ-R) | Laursen et al. 2021 |
|  | Borderline | 9-item Zanarini Rating Scale for Borderline Personality Disorder (ZAN-BPD) | Laursen et al. 2021 |
|  | Anxiety | 7-item Generalized Anxiety Disorder Questionnaire (GAD-7) | Chermahini et al. 2024 |
|  | Transdiagnostic psychopathology | 18-item general psychopathological symptom severity, subscale of HEALTH-49 | Ebert et al. 2013 |
|  |  | Clinical Global Impression (CGI) scale | Spaniel et al. 2015 |
| Recovery focused |  | 25-item Boston University Empowerment Scale (BUES), short version | Cullen et al. 2020; Lewis et al. 2020 |
|  |  | 5-item self-efficacy scale, subscale of HEALTH-49 (HEALTH-SELB) | Ebert et al. 2013 |
| Quality of life |  | 5-item psychological well-being scale, subscale of HEALTH-49 (HEALTH-WOHL) | Ebert et al. 2013 |
|  |  | 6-item EuroQol 5-Dimensions (EQ-5D-5L); utility score | Laursen et al. 2021; Lewis et al. 2020 |
|  |  | 16-item Quality of Life Enjoyment and Satisfaction Questionnaire (Q-LES-Q-Sf) | Chermahini et al. 2024 |
| (Global) functioning |  | 24-item Functional Assessment Short Test (FAST) | Faurholt-Jepsen et al. 2020 |
|  |  | Global Assessment of Functioning (GAF) | Lewis et al. 2020; Spaniel et al. 2015 |

**S4.** Descriptives of k=91 study populations included in the systematic review and meta-analysis.

|  |  | **Cases (k = 111)** | | | **Controls (k = 47)** | | |
| --- | --- | --- | --- | --- | --- | --- | --- |
|  |  | **k** | **M / % / Frequ** | **SD** | **k** | **M / %** | **SD** |
| Participants |  | 103* | 80.33 | 105.17 | 42 | 62.49 | 71.70 |
| Age |  | 92 | 40.38 | 7.70 | 32 | 421.56 | 6.92 |
| % Females |  | 92 | 56.79 |  | 32 | 55.04 |  |
| Diagnosis | Sum | 103 |  |  |  |  |  |
|  | Schizophreniform disorder | 21 (20.4%) | | | | | |
|  | Bipolar disorder | 19 (18.4%) | | | | | |
|  | Mixed sample | 19 (18.4%) | | | | | |
|  | Substance use disorder | 13 (12.6%) | | | | | |
|  | Depressive disorder | 13 (12.6%) | | | | | |
|  | Eating disorder | 4 (3.9%) | | | | | |
|  | Obsessive compulsive disorder | 3 (2.9%) | | | | | |
|  | Post-traumatic stress disorder | 3 (2.9%) | | | | | |
|  | Sleep disorder | 2 (1.9%) | | | | | |
|  | Generalized anxiety disorder | 2 (1.9%) | | | | | |
|  | Premenstrual disorder | 1 (1.0%) | | | | | |
|  | Autism spectrum disorder | 1 (1.0%) | | | | | |
| Education | Sum | 53 |  |  |  |  |  |
|  | Total years of education | 24 | 13.62 | 1.22 | 11 | 13.52 | 1.25 |
|  | % with at least highschool degree | 14 | 78.2 |  | 4 | 74.2 |  |
|  | % with college degree | 6 | 48.9 |  | 3 | 62 |  |
|  | Years of pre-university education | 4 | 13.13 | 2.18 | 1 | 11.64 |  |
|  | Years after primary school education | 3 | 5.13 | .83 | 3 | 4.9 | 1.01 |
|  | % with highschool degree | 2 | 72.1 |  | 1 | 66.2 |  |
| Type of EMA / RMBC items | Newly formulated prompts | 70 | 66.7 |  |  |  |  |
|  | Validated questionnaires | 15 | 14.3 |  |  |  |  |
|  | Combination of newly formulated prompts and validated questionnaires | 10 | 9.5 |  |  |  |  |
|  | Individualized or self designed prompts | 10 | 9.5 |  |  |  |  |
| EMA / RMBC device | Smartphone | 76 | 75.2 |  |  |  |  |
|  | Flexible (e.g., browser) | 9 | 8.9 |  |  |  |  |
|  | Mobile phone | 6 | 5.9 |  |  |  |  |
|  | Computer | 6 | 5.9 |  |  |  |  |
|  | Other | 4 | 4.0 |  |  |  |  |
| Adherence | Percentage of total measurements | 38 | 74.46 |  | 5 | 62.62 |  |
|  | Other measure of adherence | 8 |  |  | 0 |  |  |

**S5.** Additional study characteristics of studies included in meta-analysis.

| **Study** | **Inclusion criteria** | **Mean age of case group** | **Mean age of control group** | **% of female in case group** | **% female in control group** | **Years of pre-university education** | **Recruitment Setting** | **Number of study centers** |
| --- | --- | --- | --- | --- | --- | --- | --- | --- |
| Cullen et al. 2020 | Chart diagnosis of schizophrenia / schizoaffective disorder; fluent English speaking; capacity to consent; owning and texting on a cellphone; agreement to retain the same number for the duration of the study; agreement to attend the clinic for the duration of the study; the provider agreeing to participate in the study. | 48.1 (13.2) | 50.1 (14.2) | 42.9 | 41.7 | NA | Baltimore, USA | 1 |
| Chermahini et al. 2024 | ≥18 years of age; formal diagnosis of generalized anxiety disorder (GAD) according to DSM-V by a psychiatrist; consistent and reliable internet; ability to communicate and read Eng | NA | NA | NA | NA | NA | Kingston, Canada | 1 |
| Ebert et al. 2013 | ≥18 years of age; formal diagnosis of a mental disorder according to ICD-10; fluent German speaking; basic reading and writing skills; access to a computer with an internet connection. | 45 (8.8) | 45 (9.8) | 54 | 49 | NA | Marburg, Germany | 1 |
| Faurholt-Jepsen et al. 2019 | Formal diagnosis of bipolar disorder according to ICD-10; use of "Schedules for Clinical Assessments in Neuropsychiatry" (SCAN); previous treatment at the Copenhagen Clinic for Affective Disorder, Denmark. | 43 (12.4) | 43.2 (12.4) | 61.2 | 54.5 | 5.8 | Copenhagen, Denmark | 1 |
| Faurholt-Jepsen et al. 2020 | ≥18 years of age; formal diagnosis of bipolar disorder according to ICD-10; use of "Schedules for Clinical Assessments in Neuropsychiatry" (SCAN); discharged from a psychiatric hospital in the Capital Region of Denmark following an affective episode. | 41 (13.3) | 43.7 (13.7) | 51.7 | 48.33 | 5.4 | Copenhagen, Denmark | 5 |
| Gallinat et al. 2021 | F20-F29: at least 18 years old; access to a mobile phone; internet access; fluent German speaking; outpatient psychiatric provider. | 34 (10.33) | 38.2 (14.07) | 66.7 | 61.5 | NA | Heidelberg, Germany | 1 |
| Laursen et al. 2021 | ≥18 years of age; formal diagnosis of an emotionally unstable personality disorder (F60.3) according to ICD-10 by a psychiatric specialist; admission for psychiatric outpatient treatment; either self-harming or suicidal behavior within the last 3 years. | 26.4 (NA) | 27.2 (NA) | 88 | 86 | NA | Denmark | 5 |
| Lewis et al. 2020 | Aged 16-65 years; formal diagnosis of schizophrenia and related disorders according to DSM-IV; one or more psychotic episodes in the previous two years including the first psychotic episode. | 33.7 (NA) | 35.3. (NA) | 27.5 | 39 | NA | Manchester and South London, Great Britain | 2 |
| Spaniel et al. 2015 | Aged 18 to 60 years; formal diagnosis of schizophrenia or schizoaffective disorder according to ICD-10; more than one psychiatric hospitalization for psychosis before study enrolment; severity (CGI-S) ≤3 (i.e. mildly ill – clearly established symptoms with minimal, if any, distress or difficulty in social and occupational function) at baseline; on stable doses of antipsychotic medication for at least 3 months prior to study enrollment. | 36.2 (9.3) | 36.7 (9.7) | 44.6 | 43.1 | 12.4 | Prague, Czech Republic | 25 |

**Abbreviations**: ICD-10 = the International Classification of Diseases 10th Revision, DSM-IV = the Diagnostic and Statistical Manual of Mental Disorders Fourth Edition, NA = not applicable, CGI = Clinical Global Impression, SD = standard deviation

**S6.** Intervention characteristics of studies included in meta-analysis

| **Study** | **Intervention** | **RMBC element** | **Tracking items / mode** | **Number of tracking items** | **Tracking frequency** | **Study duration (weeks)** | **Alert triggered** | **Adverse event reporting** |
| --- | --- | --- | --- | --- | --- | --- | --- | --- |
| Cullen et al. 2020 | The Texting for Relapse Prevention Programme ("T4RP") aimed to identify the top five early warning signs of relapse together with the health provider. The programme sent daily text messages asking the user to determine if any of the warning signs were present. If a warning sign was present, a follow-up message was sent with specific coping strategies for that symptom. If the user did not experience any symptoms, a supportive message was sent instead. The programme also included daily questions on medication adherence and side effects, as well as inspirational quotes. | Support from healthcare providers who made personal contact within 24 hours to decide on the further course of action if users confirmed the pre-specified symptoms. | Individually formulated prompts by participants on early warning signs of relapse. | 5 items | Daily check of one of the 5 items on early warning signs and one question on adherence or coping with side effects | 24 | No | No |
| Chermahini et al. 2024 | 1: The e-CBT program consisted of previously validated modules focusing on developing coping strategies, behavior modification, cognitive restructuring, relaxation techniques, and symptom management. Each module included a weekly set of approximately 30 slides delivered through the "Online Psychotherapy Tool" (OPTT) platform. After completing the modules, participants completed homework assignments, which were reviewed by their care provider, who provided personalized feedback.  2. The check-in arm included weekly check-ins based on check-ins question prompts with their care provider. | Support from healthcare providers who gave personalised feedback to patients in both arms based on their homework and their check-in prompts. | Preformulated prompts | 9 items | Weekly | 12 | No | No |
| Ebert et al. 2013 | The internet-based maintenance therapy ("TIMT") consisting of five components: (1) a self-management module involving the development of a personal plan with individual goals and implementation intentions, (2) a web diary for participants to report on their progress in achieving their goals and setting new goals for the following week, (3) communication between participant and coach based on the web diary entries, (4) an online support group for participants to discuss their web diary entries with others, and (5) online monitoring of psychopathological symptoms. | Coach support involving weekly asynchronous written online feedback from a coach (master-level student, psychotherapist in training, certified psychotherapist) regarding participants' web diary entries | Individually defined goals and intentions for realising the goals by the participants; questionnaires on psychopathological symptoms | Individually determined | Weekly | 12 | No | No |
| Faurholt-Jepsen et al. 2019 | Smartphone-based system "Monsenso" for daily recording of symptoms, warning signs, personal prompts and free text notes. Disease activity was assessed by subjective, self-monitored measures (e.g. mood, sleep duration, activity level) and automatically recorded objective behavioural measures (e.g. phone use, social activity, physical activity, mobility, speech activity). An integrated feedback loop between the participants and clinicians included a study nurse who reviewed the collected data several times a week. If there were signs of deterioration, the study nurse contacted participants and gave advice on how to proceed (Faurholt-Jepsen et al. 2014). | Feedback-loop and study nurse support, involving several steps of action in case of signs of deterioration. If the respective lower threshold actions were not enough or possible, then 1) the participant was contacted to give advice on situation management, 2)the participant was asked to contact their usual doctor, 3) the participant was contacted by the usual doctor directly, 4) the psychiatric emergency service in Copenhagen, Denmark was contacted (Faurholt-Jepsen et al. 2014). | Preformulated prompts, individually formulated prompts by participants, free-text notes. | 9 pre-formulated items, individually determined goals, behavioural measures | Self-chosen time during the day | 36 | Yes, standard of scoring thresholds for the nurses to react | No (commented on) |
| Faurholt-Jepsen et al. 2020 | Smartphone-based system "Monsenso" for daily recording of symptoms, warning signs, personal prompts and free text notes. Disease activity was assessed by subjective, self-monitored measures (e.g. mood, sleep duration, activity level) and automatically recorded objective behavioural measures (e.g. phone use, social activity, physical activity, mobility, speech activity). An integrated feedback loop between the participants and clinicians included a study nurse who reviewed the collected data several times a week. If there were signs of deterioration, the study nurse contacted participants and gave advice on how to proceed (Faurholt-Jepsen et al. 2014). | Feedback-loop and study nurse support, involving several steps of action in case of signs of deterioration. If the respective lower threshold actions were not enough or possible, then 1) the participant was contacted to give advice on situation management, 2)the participant was asked to contact their usual doctor, 3) the participant was contacted by the usual doctor directly, 4) the psychiatric emergency service in Copenhagen, Denmark was contacted (Faurholt-Jepsen et al. 2014). | Preformulated prompts, individually formulated prompts by participants, free-text notes. | 9 pre-formulated items, individually determined goals, behavioural measures | Self-chosen time during the day | 24 | Yes, standard of scoring thresholds for the nurses to react | No (commented on) |
| Gallinat et al. 2021 | Internet-based and mobile intervention Heidelberg Internet-based Aftercare for Patients with Schizophrenia Spectrum Disorders ("HEINS"), consisting of several modules: psychoeducation about schizophrenia, an individual crisis plan, personal contact with a psychiatrist from the inpatient treatment unit via internet chat and telephone, and weekly supportive monitoring. | Alert system; in case of three unanswered monitoring questions in a row or predefined critical entries, the study doctor received a notification and called the participant to clarify whether they needed further professional support. | Short monitoring questionnaire assessing general wellbeing, amount of sleep, social contacts, medication adherence and anxiety/insecurity in the last seven days | NA | Weekly | 24 | Yes | No |
| Laursen et al. 2021 | Smartphone-based system "Monsenso" for recording of symptoms Mobile diary ("Monsenso mDiary"), containing psychoeducational material and the visualisation of participant data; passive sensor data (e.g. activity level and phone use) were collected through smartphones. | Visualisation of participant data was used in psychotherapy sessions for real time monitoring alongside treatment | Individually formulated diary entries about aspects such as emotional dysregulation, suicidal and self-harm thoughts and skill use | Individually determined diary entries; passive sensor data | Daily | 40 | No | Yes |
| Lewis et al. 2020 | Active symptom monitoring system ("ClinTouch") to encourage self-management and early intervention measures. The system provided a graphical summary of symptom fluctuation over time as a feedback to participants as well as alerts to care coordinators via the individual patient record. | When personalised early warning sign thresholds were exceeded a care coordinator was notified to make contact with participants. | Pre-formualted banching items about current symptom severity. | 12-14 items | Several times per day, 2-4 daily reminders | 12 | Yes | Yes |
| Spaniel et al. 2015 | Information Technology Aided Relapse Prevention Programme in Schizophrenia ("ITAREPS") project in which participants and family members completed early warning signs of relapse questionnaires via SMS message. If symptom thresholds were exceeded an alert system involving psychiatrists was initiated | When pre-defined symptom thresholds was exceeded a psychiatrist was notified to make contact with the patient. Early warning signs warranted an immediate 20% increase from baseline maintenance dose of antipsychotic within the next 24 hours. | Pre-formualted early warning sign questionnaire reporting proporional change compared to last week's baseline for patients and family members | 10 items | Weekly | 72 | Yes | Yes |

**S7.** Bayesian meta-analysis forest plots

1. Psychotic symptoms

**
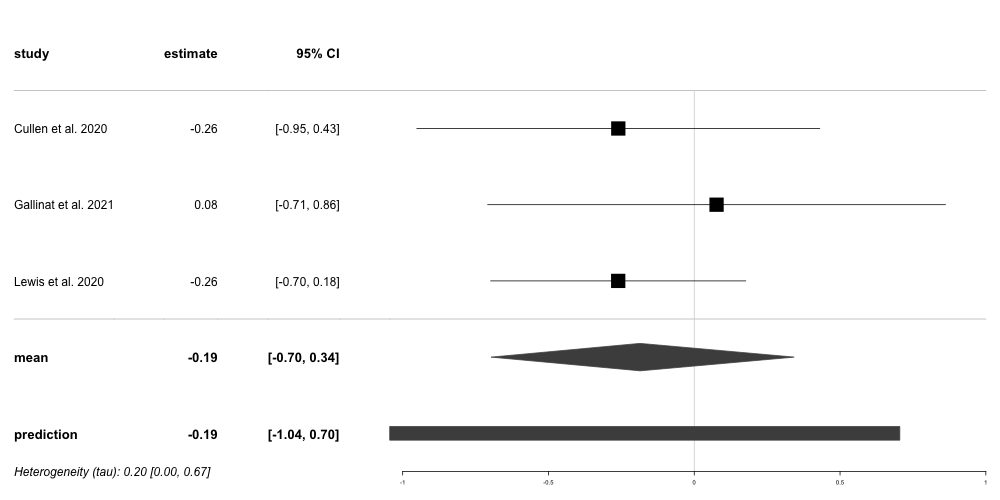
**

1. Depressive symptoms

**
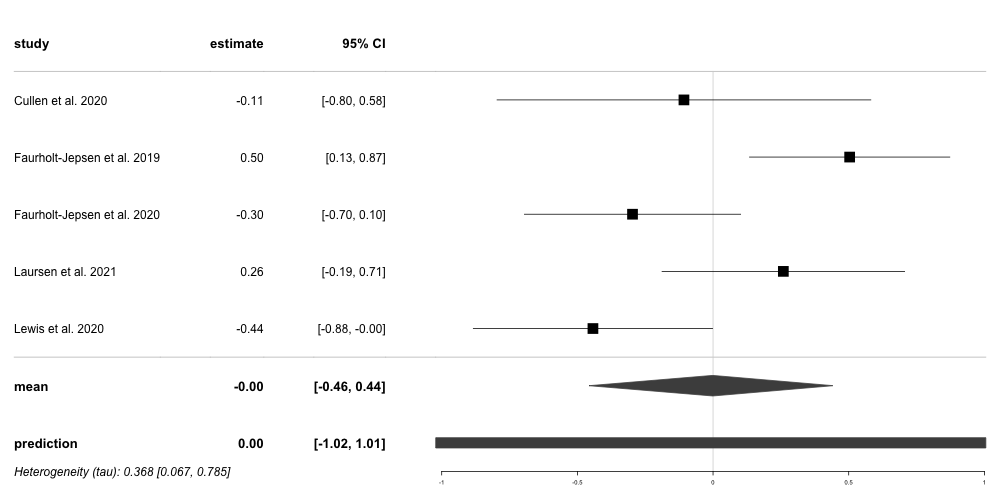
**

1. Manic symptoms

**
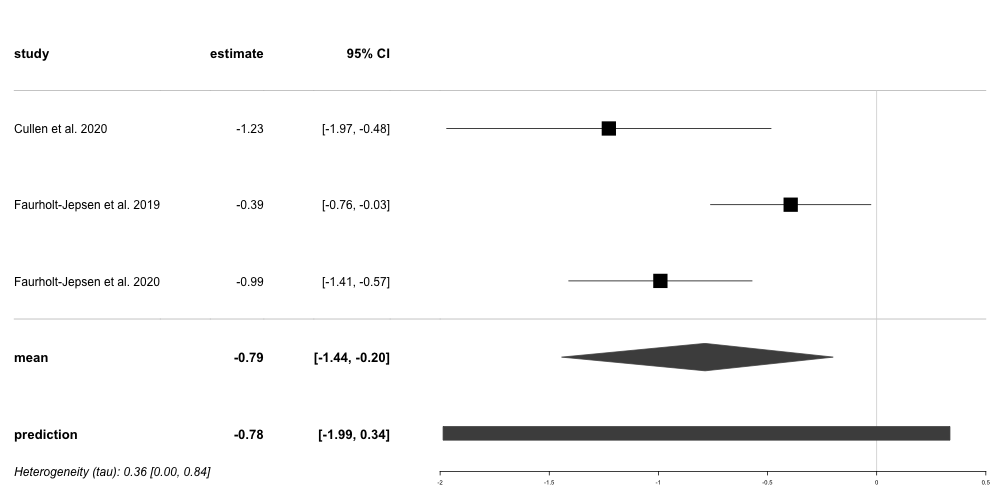
**

1. Empowerment


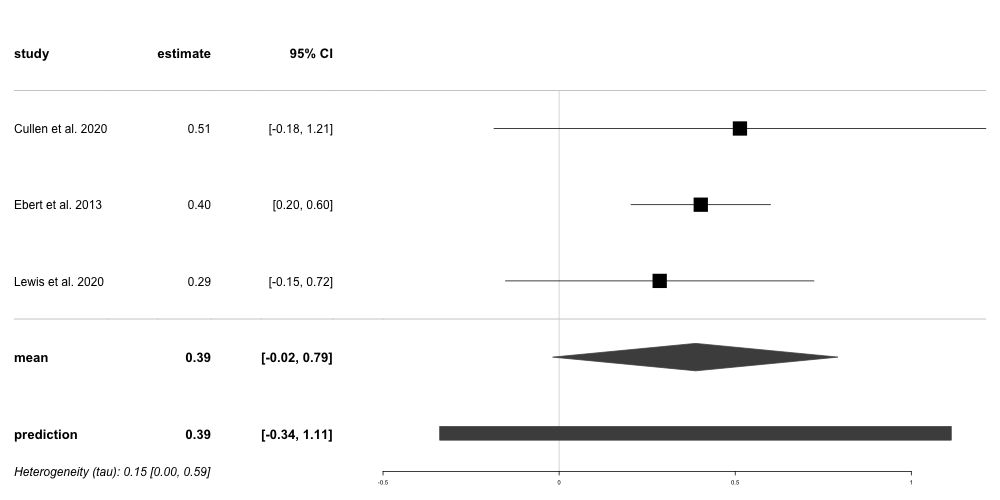


1. Quality of life


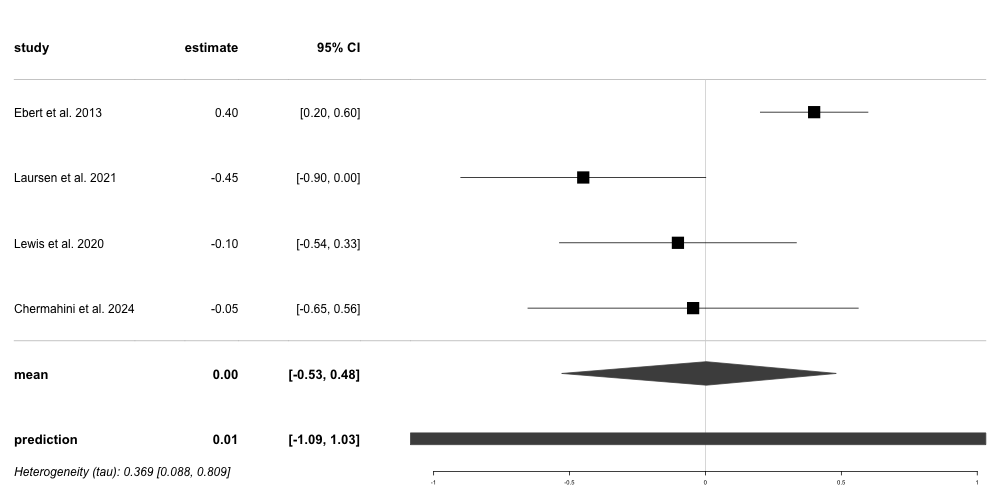


1. Functioning


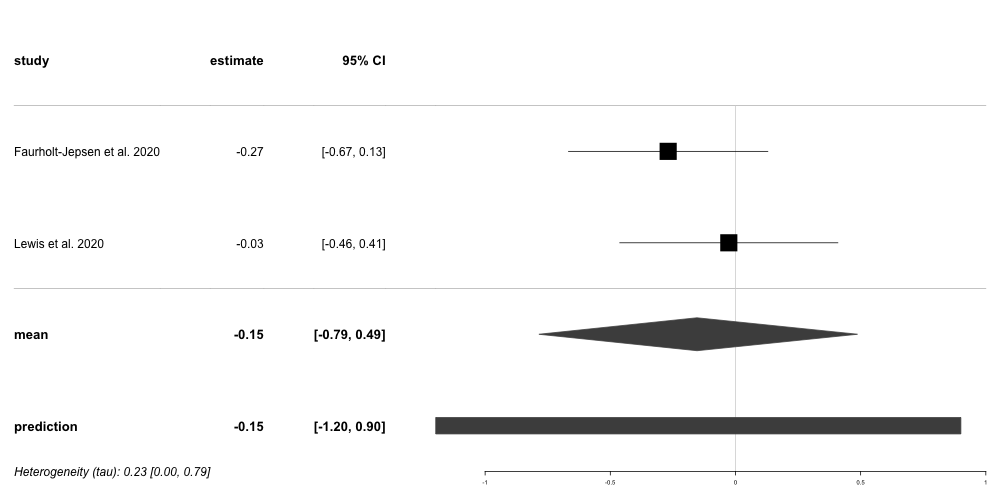


**S8.**

1. Psychotic symptoms

**
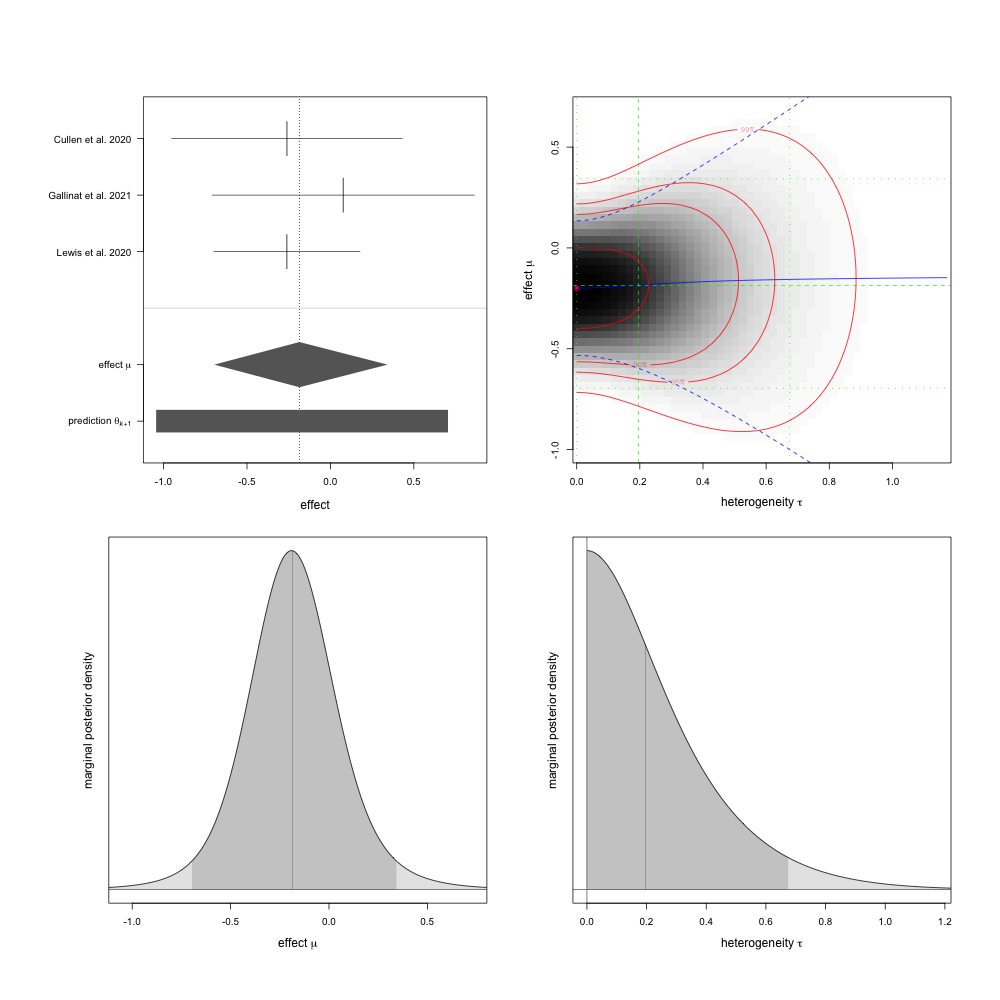
**

1. Depressive symptoms

**
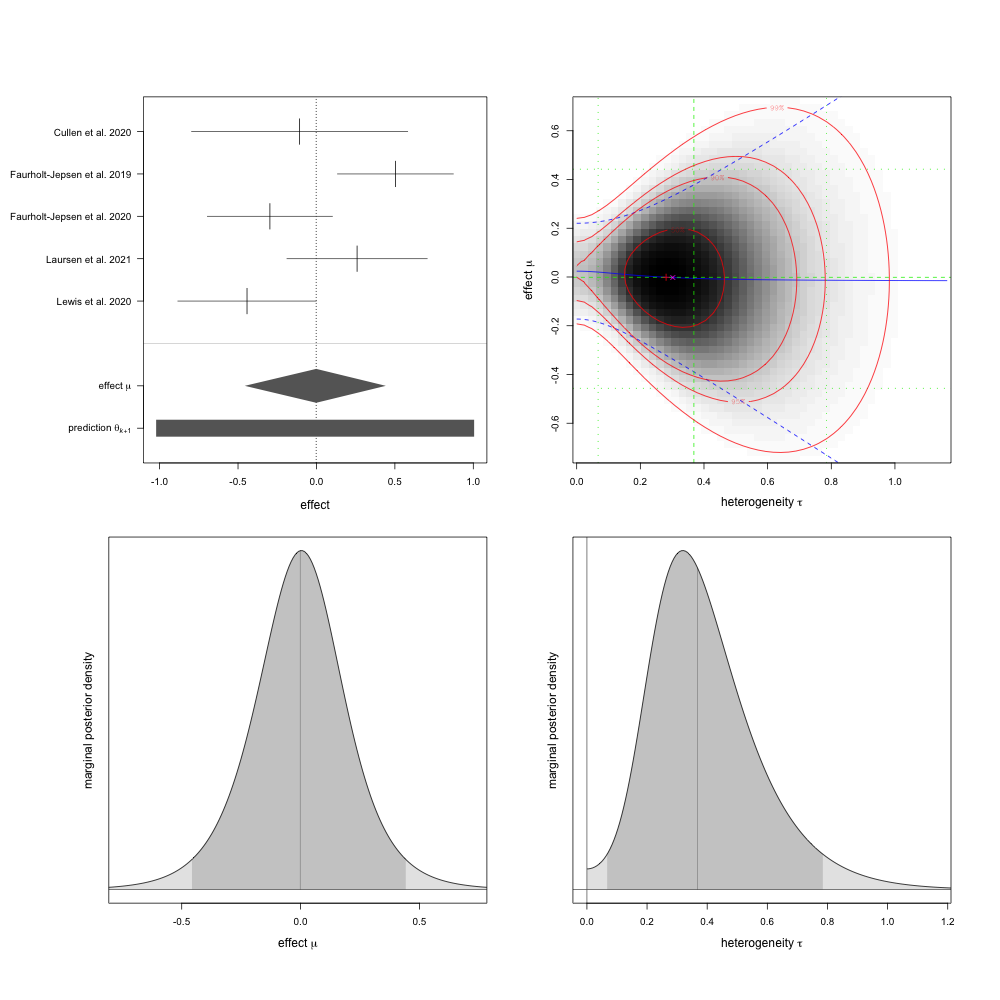
**

1. Manic symptoms

**
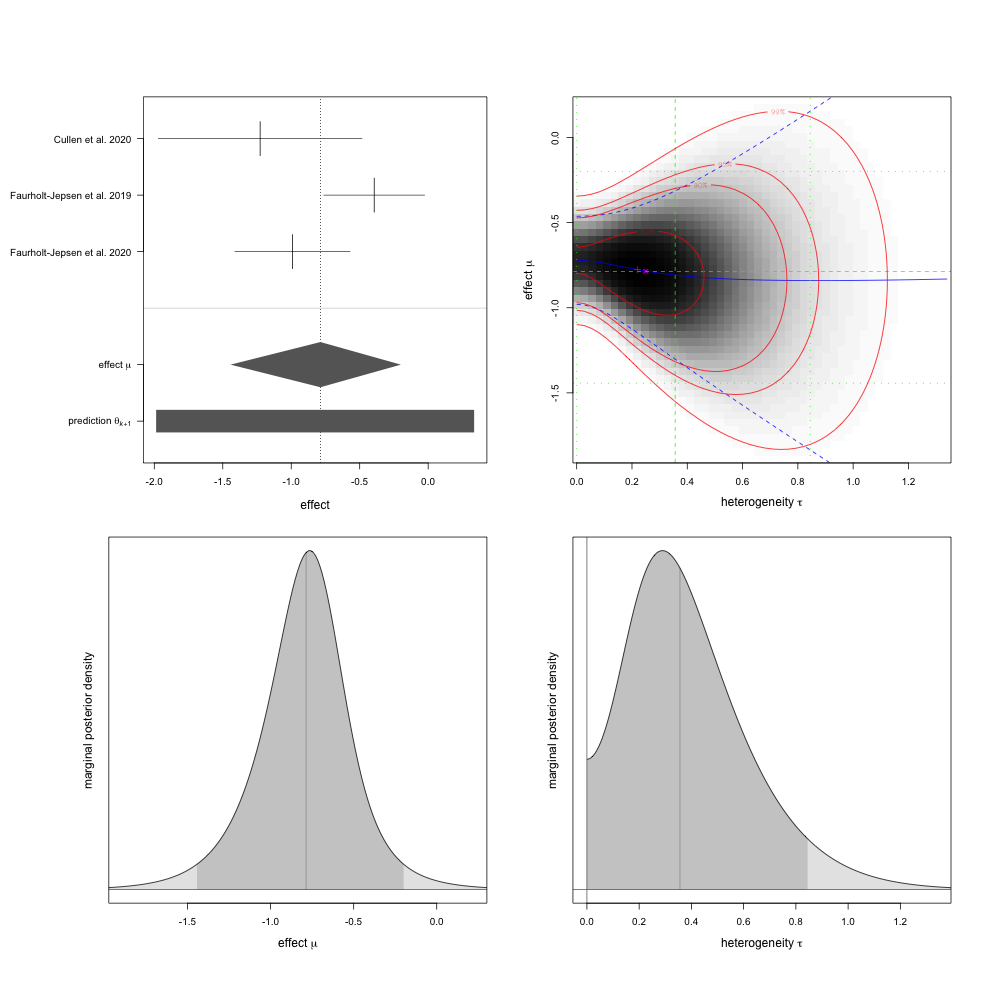
**

1. Empowerment


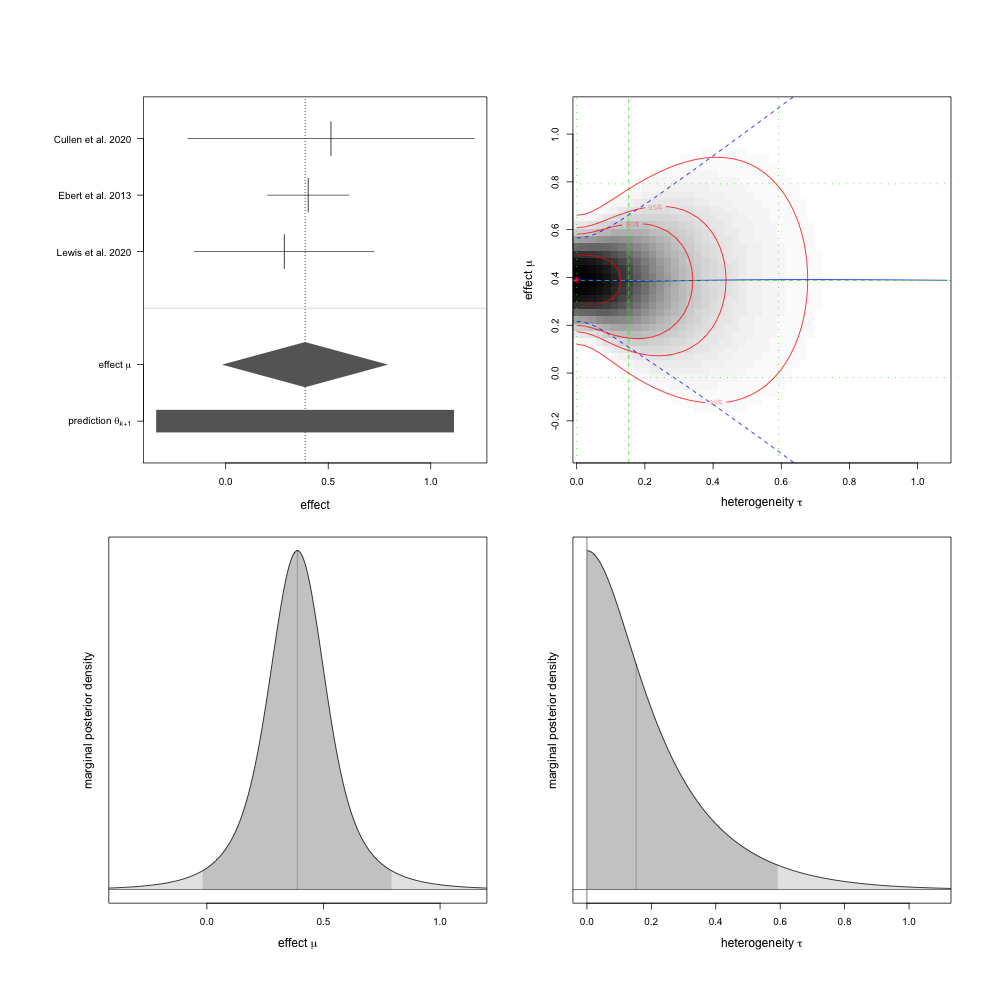


1. Quality of life


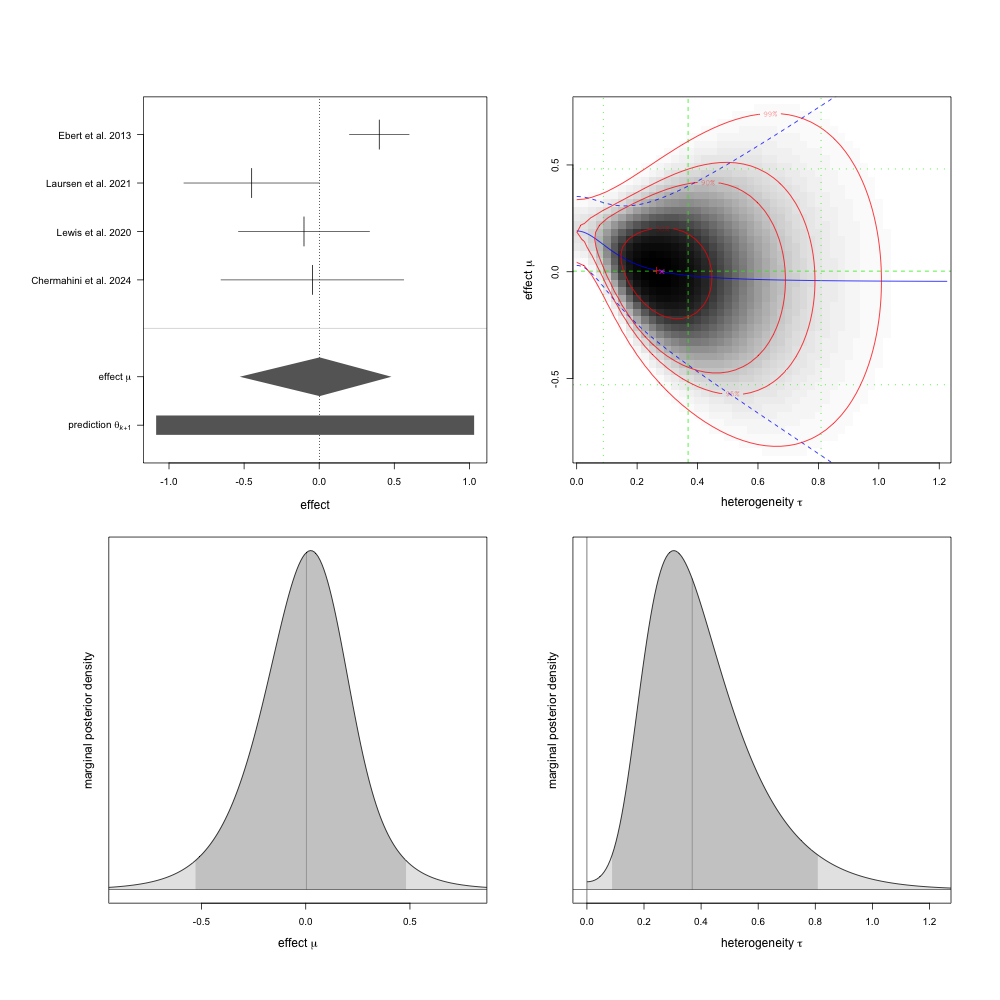


1. Functioning


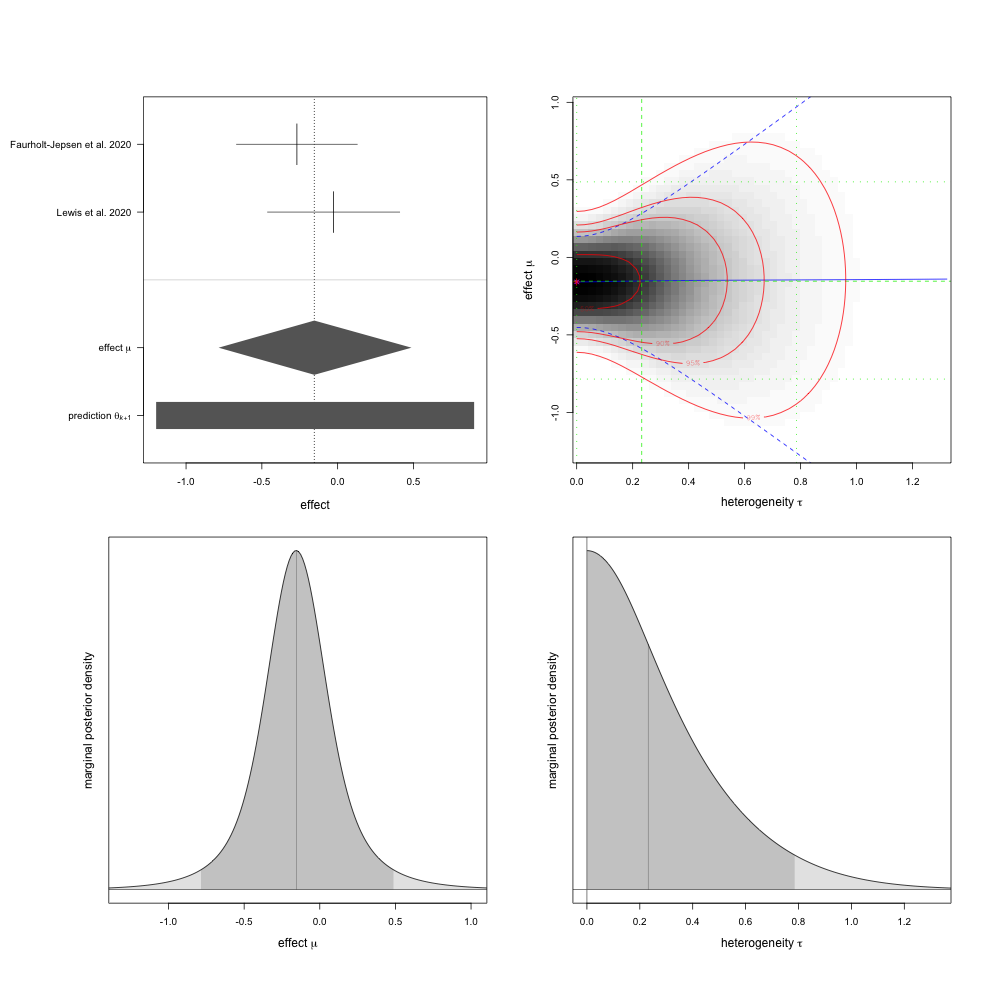


**S9.** Risk of bias assessment according to Cochrane collaboration tool 2.0. for each individual outcome at the given time point

| **Study** | **Outcome** | **Assessment Time Point** | **Weight** | **D1** | **D2** | **D3** | **D4** | **D5** | **Overall** |
| --- | --- | --- | --- | --- | --- | --- | --- | --- | --- |
| Cullen et al. 2020 | 30-item Positive and Negative Syndrome Scale (PANSS) | Baseline | 41 | 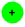 | 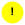 | 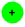 | 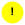 | 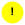 | 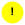 |
|  |  | 3 months | 38 | 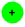 | 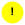 | 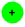 | 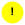 | 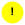 | 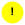 |
|  |  | 6 months | 37 | 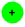 | 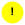 | 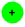 | 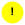 | 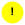 | 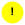 |
|  | 10-item Montgomery-Asberg Depression Rating Scale (MADRS) | Baseline | 41 | 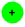 | 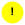 | 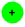 | 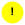 | 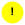 | 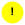 |
|  |  | 3 months | 38 | 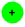 | 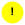 | 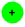 | 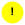 | 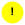 | 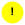 |
|  |  | 6 months | 37 | 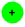 | 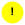 | 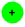 | 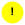 | 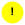 | 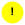 |
|  | 11-item Young Mania Rating Scale (YMRS) | Baseline | 41 | 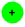 | 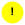 | 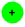 | 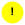 | 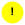 | 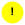 |
|  |  | 3 months | 38 | 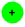 | 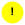 | 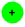 | 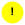 | 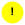 | 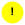 |
|  |  | 6 months | 37 | 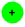 | 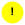 | 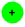 | 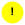 | 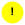 | 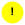 |
|  | 24-item Recovery Assessment Scale Revised (RAS-R) | Baseline | 41 | 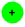 | 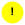 | 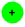 | 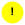 | 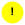 | 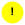 |
|  |  | 3 months | 38 | 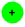 | 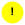 | 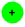 | 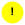 | 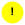 | 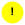 |
|  |  | 6 months | 37 | 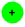 | 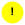 | 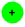 | 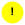 | 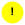 | 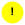 |
|  | Number of hospitalisations (n=%) | Baseline | 41 | 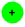 | 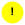 | 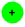 | 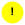 | 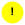 | 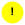 |
|  |  | 3 months | 38 | 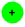 | 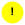 | 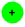 | 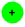 | 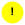 | 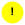 |
|  |  | 6 months | 37 | 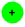 | 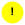 | 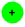 | 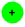 |  |  |
|  | Number of psychiatric ER visits (n=%) | Baseline | 41 |  |  |  |  |  |  |
|  |  | 3 months | 38 |  |  |  |  |  |  |
|  |  | 6 months | 37 |  |  |  |  |  |  |
|  | Number of intensive outpatient program referrals (n=%) | Baseline | 41 |  |  |  |  |  |  |
|  |  | 3 months | 38 |  |  |  |  |  |  |
|  |  | 6 months | 37 |  |  |  |  |  |  |
|  | 4-item Brief Adherence Rating Scale (BARS), Oral medication adherence (>=90%, n=%) | Baseline | 30 |  |  |  |  |  |  |
|  |  | 3 months | 38 |  |  |  |  |  |  |
|  |  | 6 months | 34 |  |  |  |  |  |  |
|  | Injectable medication adherence (>=90%, n=%) | Baseline | 4 |  |  |  |  |  |  |
|  |  | 3 months | 9 |  |  |  |  |  |  |
|  |  | 6 months | 7 |  |  |  |  |  |  |
|  | 25-item Boston University Empowerment Scale (BUES), short version | Baseline | 40 |  |  |  |  |  |  |
|  |  | 3 months | 38 |  |  |  |  |  |  |
|  |  | 6 months | 37 |  |  |  |  |  |  |
|  | Score on improving communication between patients and providers | Baseline | 41 |  |  |  |  |  |  |
|  |  | 3 months | 38 |  |  |  |  |  |  |
|  |  | 6 months | 37 |  |  |  |  |  |  |
| Ebert et al. 2013 | 18-item general psychopathological symptom severity, subscale of HEALTH-49 | Admission | 400 |  |  |  |  |  |  |
|  |  | Baseline | 400 |  |  |  |  |  |  |
|  |  | 3 months | 400 |  |  |  |  |  |  |
|  |  | 12 months | 400 |  |  |  |  |  |  |
|  | 5-item psychological well-being scale, subscale of HEALTH-49 (HEALTH-WOHL) | Admission | 400 |  |  |  |  |  |  |
|  |  | Baseline | 400 |  |  |  |  |  |  |
|  |  | 3 months | 400 |  |  |  |  |  |  |
|  |  | 12 months | 400 |  |  |  |  |  |  |
|  | Positive affect, 10-item Positive And Negative Affect Schedule (PANAS) | Admission | 400 |  |  |  |  |  |  |
|  |  | Baseline | 400 |  |  |  |  |  |  |
|  |  | 3 months | 400 |  |  |  |  |  |  |
|  |  | 12 months | 400 |  |  |  |  |  |  |
|  | Negative affect, 10-item Positive And Negative Affect Schedule (PANAS) | Admission | 400 |  |  |  |  |  |  |
|  |  | Baseline | 400 |  |  |  |  |  |  |
|  |  | 3 months | 400 |  |  |  |  |  |  |
|  |  | 12 months | 400 |  |  |  |  |  |  |
|  | 7-item interpersonal problems scale, subscale of HEALTH-49 (HEALTH-INT ) | Admission | 400 |  |  |  |  |  |  |
|  |  | Baseline | 400 |  |  |  |  |  |  |
|  |  | 3 months | 400 |  |  |  |  |  |  |
|  |  | 12 months | 400 |  |  |  |  |  |  |
|  | 5-item self-efficacy scale, subscale of HEALTH-49 (HEALTH-SELB) | Admission | 400 |  |  |  |  |  |  |
|  |  | Baseline | 400 |  |  |  |  |  |  |
|  |  | 3 months | 400 |  |  |  |  |  |  |
|  |  | 12 months | 400 |  |  |  |  |  |  |
|  | 27-item Emotion Regulation Skills Questionnaire (ERSQ) | Admission | 400 |  |  |  |  |  |  |
|  |  | Baseline | 400 |  |  |  |  |  |  |
|  |  | 3 months | 400 |  |  |  |  |  |  |
|  |  | 12 months | 400 |  |  |  |  |  |  |
| Faurholt-Jepsen et al. 2019 | 17-item Hamilton Depression Rating Scale (HDRS) | Baseline | 129 |  |  |  |  |  |  |
|  |  | 9 months | 129 |  |  |  |  |  |  |
|  | 11-item Young Mania Rating Scale (YMRS) | Baseline | 129 |  |  |  |  |  |  |
|  |  | 9 months | 129 |  |  |  |  |  |  |
| Faurholt-Jepsen et al. 2020 | 17-item Hamilton Depression Rating Scale (HDRS) | Baseline | 98 |  |  |  |  |  |  |
|  |  | 6 months | 98 |  |  |  |  |  |  |
|  | 11-item Young Mania Rating Scale (YMRS) | Baseline | 98 |  |  |  |  |  |  |
|  |  | 6 months | 98 |  |  |  |  |  |  |
|  | 24-item Functional Assessment Short Test (FAST) | Baseline | 98 |  |  |  |  |  |  |
|  |  | 6 months | 98 |  |  |  |  |  |  |
|  | Readmission (n) | 6 months | 98 |  |  |  |  |  |  |
|  | Duration of readmission (days) | 6 months | 98 |  |  |  |  |  |  |
|  | Duration of days to readmission (days) | 6 months | 98 |  |  |  |  |  |  |
| Gallinat et al. 2021 | 30-item Positive and Negative Syndrome Scale (PANSS) | Baseline | 25 |  |  |  |  |  |  |
|  |  | 6 months | 25 |  |  |  |  |  |  |
| Laursen et al. 2021 | 6-item EuroQol 5-Dimensions (EQ-5D-5L); mobility | Baseline | 78 |  |  |  |  |  |  |
|  |  | 12 weeks | 78 |  |  |  |  |  |  |
|  | 6-item EuroQol 5-Dimensions (EQ-5D-5L); self-care | Baseline | 78 |  |  |  |  |  |  |
|  |  | 12 weeks | 78 |  |  |  |  |  |  |
|  | 6-item EuroQol 5-Dimensions (EQ-5D-5L); usual activities | Baseline | 78 |  |  |  |  |  |  |
|  |  | 12 weeks | 78 |  |  |  |  |  |  |
|  | 6-item EuroQol 5-Dimensions (EQ-5D-5L); pain or discomfort | Baseline | 78 |  |  |  |  |  |  |
|  |  | 12 weeks | 78 |  |  |  |  |  |  |
|  | 6-item EuroQol 5-Dimensions (EQ-5D-5L); anxiety or depression | Baseline | 78 |  |  |  |  |  |  |
|  |  | 12 weeks | 78 |  |  |  |  |  |  |
|  | 6-item EuroQol 5-Dimensions (EQ-5D-5L); utility score | Baseline | 78 |  |  |  |  |  |  |
|  |  | 12 weeks | 78 |  |  |  |  |  |  |
|  | QALY gain | NA | 78 |  |  |  |  |  |  |
|  | 9-item Zanarini Rating Scale for Borderline Personality Disorder (ZAN-BPD) | Baseline | 78 |  |  |  |  |  |  |
|  |  | 12 weeks | 78 |  |  |  |  |  |  |
|  | 9-item Patient Health Questionnaire (PHQ-9) | Baseline | 78 |  |  |  |  |  |  |
|  |  | 12 weeks | 78 |  |  |  |  |  |  |
|  | 4-item Suicidal Behaviors Questionnaire-Revised (SBQ-R) | Baseline | 78 |  |  |  |  |  |  |
|  |  | 12 weeks | 78 |  |  |  |  |  |  |
|  | Readmission rate (n) | 30 days before baseline | 78 |  |  |  |  |  |  |
|  |  | 30 days after baseline | 78 |  |  |  |  |  |  |
|  | General practice contacts (mean) | 12 months before baseline | 78 |  |  |  |  |  |  |
|  |  | 12 months after baseline | 78 |  |  |  |  |  |  |
|  | Treatment compliance, participants, who never start treatment (n) | NA | 78 |  |  |  |  |  |  |
|  | Treatment compliance, number of treatment days for participants starting treatment (mean, SE) | NA | 78 |  |  |  |  |  |  |
|  | Skill recordings per patient for all participants | NA | 78 |  |  |  |  |  |  |
|  | Skill recordings per patient for participants starting treatment | NA | 78 |  |  |  |  |  |  |
|  | Skill recordings per week for participants starting treatment | NA | 78 |  |  |  |  |  |  |
| Lewis et al. 2020 | 30-item Positive and Negative Syndrome Scale (PANSS); total | Baseline | 81 |  |  |  |  |  |  |
|  |  | 6 weeks | 81 |  |  |  |  |  |  |
|  |  | 12 weeks | 81 |  |  |  |  |  |  |
|  | 30-item Positive and Negative Syndrome Scale (PANSS); positive scale | Baseline | 81 |  |  |  |  |  |  |
|  |  | 6 weeks | 81 |  |  |  |  |  |  |
|  |  | 12 weeks | 81 |  |  |  |  |  |  |
|  | 30-item Positive and Negative Syndrome Scale (PANSS); negative scale | Baseline | 81 |  |  |  |  |  |  |
|  |  | 6 weeks | 81 |  |  |  |  |  |  |
|  |  | 12 weeks | 81 |  |  |  |  |  |  |
|  | 30-item Positive and Negative Syndrome Scale (PANSS); general psychopathology | Baseline | 81 |  |  |  |  |  |  |
|  |  | 6 weeks | 81 |  |  |  |  |  |  |
|  |  | 12 weeks | 81 |  |  |  |  |  |  |
|  | 28-item Boston University Empowerment Scale (BUES) | Baseline | 81 |  |  |  |  |  |  |
|  |  | 6 weeks | 81 |  |  |  |  |  |  |
|  |  | 12 weeks | 81 |  |  |  |  |  |  |
|  | 6-item EuroQol 5-Dimensions (EQ-5D-5L); quality of life | Baseline | 81 |  |  |  |  |  |  |
|  |  | 6 weeks | 81 |  |  |  |  |  |  |
|  |  | 12 weeks | 81 |  |  |  |  |  |  |
|  | 9-item Calgary Depression Scale (CDS) | Baseline | 81 |  |  |  |  |  |  |
|  |  | 6 weeks | 81 |  |  |  |  |  |  |
|  |  | 12 weeks | 81 |  |  |  |  |  |  |
|  | Global Assessment of Functioning (GAF) | Baseline | 81 |  |  |  |  |  |  |
|  |  | 6 weeks | 81 |  |  |  |  |  |  |
|  |  | 12 weeks | 81 |  |  |  |  |  |  |
| Spaniel et al. 2015 | Hospitalisations (n) | 18 months | 146 |  |  |  |  |  |  |
|  | Number of inpatient days (mean) | 18 months | 146 |  |  |  |  |  |  |
|  | Global Assessment of Functioning (GAF) | Baseline | 146 |  |  |  |  |  |  |
|  | Clinical Global Impression (CGI) scale | Baseline | 146 |  |  |  |  |  |  |
| Chermahini et al. 2024 | 7-item Generalized Anxiety Disorder Questionnaire (GAD-7) | Baseline | 95 |  |  |  |  |  |  |
|  |  | 6 weeks | 72 |  |  |  |  |  |  |
|  |  | 12 weeks | 51 |  |  |  |  |  |  |
|  | 16-item Quality of Life Enjoyment and Satisfaction Questionnaire (Q-LES-Q-Sf) | Baseline | 88 |  |  |  |  |  |  |
|  |  | 6 weeks | 63 |  |  |  |  |  |  |
|  |  | 12 weeks | 42 |  |  |  |  |  |  |
|  | 42-item Depression Anxiety Stress Scale (DASS-42) - Depression | Baseline | 94 |  |  |  |  |  |  |
|  |  | 6 weeks | 70 |  |  |  |  |  |  |
|  |  | 12 weeks | 50 |  |  |  |  |  |  |
|  | 42-item Depression Anxiety Stress Scale (DASS-42) - Anxiety | Baseline | 95 |  |  |  |  |  |  |
|  |  | 6 weeks | 69 |  |  |  |  |  |  |
|  |  | 12 weeks | 52 |  |  |  |  |  |  |
|  | 42-item Depression Anxiety Stress Scale (DASS-42) - Stress | Baseline | 95 |  |  |  |  |  |  |
|  |  | 6 weeks | 68 |  |  |  |  |  |  |
|  |  | 12 weeks | 52 |  |  |  |  |  |  |
|  | Low risk | | | | | | | | |
|  | Some concerns | | | | | | | | |
|  | High risk | | | | | | | | |

D1: Randomization process, D2 Deviations from the intended outcome, D3: Missing outcome data, D4: Measurement of the outcome, D5: Section of the reported result

**S10.** Diagnostic plots of linear regression model

**S11.** R code for meta analysis

```{r setup, include=FALSE}

knitr::opts_chunk$set(echo = TRUE)

# numbers >= 10^5 will be denoted in scientific notation,

## and rounded to 2 digits

options(scipen = 1, digits = 2)

f_pvalue = function(p.value, symbol = "="){

p.value <- round(p.value, digits = 3)

if (p.value == 0) {

return("p < .001")

} else {

return(paste0("p", symbol, round(p.value, digits = 3)))

}

}

#load all necessary packages

library(readxl)

library(googlesheets4)

library(tidyverse)

library(meta)

library(metafor)

```

```{r meta analysis, include=F}

# Load data

df <- read.csv("~/Documents/GitHub/RMBC_meta/SysRevdataextraction - Data extraction.csv")

# Define a function to filter and clean the dataframe

filter_and_clean <- function(data, constructs) {

data %>%

filter(

!is.na(Construct),

`timepoint_def` == "End of intervention",

Design == "RCT",

Construct %in% constructs

) %>%

mutate(

effect_size = (mean_case - mean_control) / sqrt(((ncases_by_outcome - 1) * sd_case^2 + (ncontrol_by_outcome - 1) * sd_control^2) / (ncases_by_outcome + ncontrol_by_outcome - 2)),

var_effect_size = (ncases_by_outcome + ncontrol_by_outcome) / (ncases_by_outcome * ncontrol_by_outcome) + effect_size^2 / (2 * (ncases_by_outcome + ncontrol_by_outcome)),

std_error = sqrt(var_effect_size)

)

}

# Meta-analysis function

conduct_meta_forest <- function(subgroup_name, data) {

if (nrow(data) == 0) {

cat(paste("No data available for", subgroup_name, "\n"))

return(NULL)

}

meta_res <- tryCatch({

metacont(

n.e = data$ncases_by_outcome,

mean.e = data$mean_case,

sd.e = data$sd_case,

n.c = data$ncontrol_by_outcome,

mean.c = data$mean_control,

sd.c = data$sd_control,

studlab = data$study,

sm = "SMD",

method.smd = "Hedges",

method.tau = "REML"

)

}, error = function(e) {

cat(paste("Error in metacont for", subgroup_name, ":", e$message, "\n"))

return(NULL)

})

if (is.null(meta_res)) {

return(NULL)

}

# Update meta-analysis object with subgroups

meta_res <- tryCatch({

update(meta_res, subgroup = data$Construct, tau.common = FALSE)

}, error = function(e) {

cat(paste("Error in update for", subgroup_name, ":", e$message, "\n"))

return(meta_res) # Return the original meta_res if update fails

})

# Test for publication bias

result_metabias <- metabias(meta_res)

print(result_metabias)

# Save the forest plot with subgroups

png(paste0("~/Desktop/forest_plot_", gsub(" ", "_", subgroup_name), ".png"), width = 3500, height = 1600, res = 300)

forest(meta_res, xlab = "Hedges' g", col.box = "royalblue", col.summary = "darkblue", main = paste("Forest Plot for", subgroup_name))

dev.off()

# Extract and print p-value for the SMD

p_value <- summary(meta_res)$pval.random

formatted_p_value <- format.pval(p_value)

cat(paste("Meta-analysis for", subgroup_name, ": p-value =", formatted_p_value, "\n"))

# Calculate t-test for each study

t_test_results <- data %>%

rowwise() %>%

mutate(

t_stat = (mean_case - mean_control) / sqrt((sd_case^2 / ncases_by_outcome) + (sd_control^2 / ncontrol_by_outcome)),

p_value = 2 * pt(-abs(t_stat), df = ncases_by_outcome + ncontrol_by_outcome - 2)

)

print(t_test_results)

}

# Apply functions

df_psych <- filter_and_clean(df, c("Psychotic symptoms"))

conduct_meta_forest("Psychotic symptoms", df_psych)

df_depr <- filter_and_clean(df, c("Depressive symptoms"))

conduct_meta_forest("Depressive symptoms", df_depr)

df_manic <- filter_and_clean(df, c("Manic symptoms"))

conduct_meta_forest("Manic symptoms", df_manic)

df_trans <- filter_and_clean(df, c("Transdiagnostic"))

conduct_meta_forest("Transdiagnostic", df_trans)

df_empower <- filter_and_clean(df, c("Empowerment"))

conduct_meta_forest("Empowerment", df_empower)

df_qol <- filter_and_clean(df, c("Quality of life"))

conduct_meta_forest("QoL", df_qol)

df_fun <- filter_and_clean(df, c("Functioning"))

conduct_meta_forest("Functioning", df_fun)

```

```{r bayesian, include=FALSE}

library(bayesmeta)

library(hexbin)

# List of constructs to analyze

constructs <- c("Manic symptoms", "Depressive symptoms", "Psychotic symptoms", "Recovery", "Quality of life", "Functioning")

# Loop through each construct

for (construct in constructs) {

# Filter and clean the data for the current construct

df_construct <- filter_and_clean(df, construct)

# Check if the data frame is empty

if (nrow(df_construct) == 0) {

cat("No data available for", construct, "\n")

} else {

# Perform Bayesian meta-analysis

bayes_analysis <- bayesmeta(

y = df_construct$effect_size,

sigma = df_construct$std_error,

labels = df_construct$study,

mu.prior.mean = 0,

mu.prior.sd = 4,

tau.prior = function(t) { dhalfnormal(t, scale = 0.5) }

)

# Print the results for the current construct

print(bayes_analysis)

# Generate and save the forest plot without shrinkage estimates

png(paste0("~/Desktop/bm_", gsub(" ", "_", tolower(construct)), "_fp.png"), width = 1000, height = 500)

forestplot(bayes_analysis, shrink = FALSE)

dev.off()

# Generate and save the plots including the prior

png(paste0("~/Desktop/bm_", gsub(" ", "_", tolower(construct)), "_plots.png"), width = 1000, height = 1000)

par(mfrow = c(2, 2), mar = c(2, 5, 4, 2) + 0.1, oma = c(6, 4, 4, 2), cex.lab = 0.7)

plot(bayes_analysis, which = 1, main = "") # Forest plot

plot(bayes_analysis, which = 2, main = "") # Funnel plot with prior

plot(bayes_analysis, which = 3, main = "") # Marginal posterior density for effect

plot(bayes_analysis, which = 4, main = "") # Marginal posterior density for heterogeneity

dev.off()

}

}

**S12.** R code for linear regression model

```{r setup, include=FALSE}

knitr::opts_chunk$set(echo = TRUE)

# Load df

library(readr)

df <- read_csv("~/Documents/GitHub/RMBC_meta/ma_regression.csv")

```

```{r eda, include=FALSE}

# Exploratory Data Analysis

summary(df)

# Using histograms to check distribution of variables

par(mfrow=c(1,3))

hist(df$prompts_per_day, main="Prompts per day", xlab="Prompts per day", col="grey", border="black")

hist(df$n_items, main="Number of Tracking Items", xlab="Number of tracking items", col="grey", border="black")

hist(df$response_rate, main="Response rate (%)", xlab="Response rate (%)", col="grey", border="black")

# Box plotx to detect outliers

par(mfrow=c(1,3))

boxplot(df$prompts_per_day, main="Prompts per day", ylab="Prompts per day", col="grey", border="black")

boxplot(df$n_items, main="Number of tracking items", ylab="Number of tracking items", col="grey", border="black")

boxplot(df$response_rate, main="Response rate (%)", ylab="Response rate (%)", col="grey", border="black")

# Scatter plots to visualise the relationship between the variables

par(mfrow=c(1,2), mar=c(5.1, 4.1, 6.1, 2.1))

plot(df$prompts_per_day, df$response_rate, main="Response rate (%) vs.\nprompts per day",

xlab="Prompts per day", ylab="Response rate (%)", pch=19, col="black")

plot(df$n_items, df$response_rate, main="Response rate (%) vs.\nnumber of tracking items",

xlab="Number of tracking items", ylab="Response rate (%)", pch=19, col="black")

# Resetting to default plotting settings

par(mfrow=c(1,1), mar=c(5.1, 4.1, 4.1, 2.1))

# Correlation matrix

cor(df[, c("prompts_per_day", "n_items", "response_rate")], use="complete.obs")

```

```{r assumptions, include=FALSE}

# Check for Linearity

par(mfrow = c(1, 2))

par(family = "Times New Roman")

plot(df$prompts_per_day, df$response_rate, main="Prompts/day vs. response rate",

xlab="Prompts/d (n)", ylab="Response rate (%)",

cex.main=1)

plot(df$n_items, df$response_rate, main="Tracking items vs. response rate",

xlab="Tracking items (n)", ylab="Response rate (%)",

cex.main=1)

# Check dependent variable for normality

par(mfrow = c(1, 2))

par(family = "Times New Roman")

hist(df$response_rate, main="Response rate (%)", xlab="Response rate (%)", col="grey", border="black")

qqnorm(df$response_rate, main="QQ-plot for Response Rate")

qqline(df$response_rate)

# Check for Multicollinearity using the Variance Inflation Factor (VIF)

library(car)

# Assuming df has only the necessary columns for the model

vif_model <- lm(response_rate ~ prompts_per_day + n_items, data=df)

vif(vif_model)

# Check for Independence of Residuals Durbin-Watson test

install.packages("lmtest")

library(lmtest)

dwtest(vif_model)

```

```{r lm, include=FALSE}

# Creating log

df$log_prompts_per_day <- log(df$prompts_per_day + 1) # Add +1 to avoid log(0) errors

df$log_n_items <- log(df$n_items + 1)

# Fit linear regression model to the log transformed predictors

model_lm2 <- lm(response_rate ~ log_prompts_per_day + log_n_items, data=df)

summary(model_lm2)

# Creating a bubble plot

bubble_plot_lm <- ggplot(df, aes(x=log_prompts_per_day, y=response_rate, size=log_n_items)) +

geom_point(color="blue", alpha=0.6, show.legend=FALSE) +

geom_smooth(method="lm", se=TRUE, color="black") +

scale_size_continuous(range = c(1, 10)) +

theme_minimal() +

theme(

text = element_text(family="Arial", size=12),

axis.title = element_text(size=12),

axis.text = element_text(size=12),

legend.position="right",

legend.title.align=0.5,

panel.grid.major = element_blank(),

panel.grid.minor = element_blank(),

panel.border = element_rect(colour = "black", fill=NA, size=1)

) +

labs(

x="Log prompts per day",

y="Response rate (%)"

) +

guides(size=FALSE) # This also removes the size legend completely

print(bubble_plot_lm)

# Diagnostic plots

par(mfrow=c(2, 2))

par(family = "Arial")

plot(model_lm2)

```
